# Neonatal brain metabolite concentrations: Associations with age, sex, and developmental outcomes

**DOI:** 10.1371/journal.pone.0243255

**Published:** 2020-12-17

**Authors:** Emily C. Merz, Catherine Monk, Ravi Bansal, Siddhant Sawardekar, Seonjoo Lee, Tianshu Feng, Marisa Spann, Sophie Foss, Laraine McDonough, Elizabeth Werner, Bradley S. Peterson

**Affiliations:** 1 Department of Psychology, Colorado State University, Fort Collins, CO, United States of America; 2 Department of Psychiatry, Columbia University Irving Medical Center, New York, NY, United States of America; 3 Department of Obstetrics and Gynecology, Columbia University Irving Medical Center, New York, NY, United States of America; 4 New York State Psychiatric Institute, New York, NY, United States of America; 5 Department of Pediatrics, Children’s Hospital Los Angeles and the University of Southern California, Los Angeles, CA, United States of America; 6 Institute for the Developing Mind, Children’s Hospital Los Angeles, Los Angeles, CA, United States of America; 7 Department of Psychology, Brooklyn College, New York, New York, United States of America; 8 City University of New York Graduate Center, New York, New York, United States of America; 9 Division of Child and Adolescent Psychiatry, Department of Psychiatry, Keck School of Medicine, University of Southern California, Los Angeles, CA, United States of America; Western University, CANADA

## Abstract

Age and sex differences in brain metabolite concentrations in early life are not well understood. We examined the associations of age and sex with brain metabolite levels in healthy neonates, and investigated the associations between neonatal brain metabolite concentrations and developmental outcomes. Forty-one infants (36–42 gestational weeks at birth; 39% female) of predominantly Hispanic/Latina mothers (mean 18 years of age) underwent MRI scanning approximately two weeks after birth. Multiplanar chemical shift imaging was used to obtain voxel-wise maps of N-acetylaspartate (NAA), creatine, and choline concentrations across the brain. The Bayley Scales of Infant and Toddler Development, a measure of cognitive, language, and motor skills, and mobile conjugate reinforcement paradigm, a measure of learning and memory, were administered at 4 months of age. Findings indicated that postmenstrual age correlated positively with NAA concentrations in multiple subcortical and white matter regions. Creatine and choline concentrations showed similar but less pronounced age related increases. Females compared with males had higher metabolite levels in white matter and subcortical gray matter. Neonatal NAA concentrations were positively associated with learning and negatively associated with memory at 4 months. Age-related increases in NAA, creatine, and choline suggest rapid development of neuronal viability, cellular energy metabolism, and cell membrane turnover, respectively, during early life. Females may undergo earlier and more rapid regional developmental increases in the density of viable neurons compared to males.

## Introduction

Brain development during the perinatal period and in the first few years of life is increasingly being studied. A variety of magnetic resonance imaging (MRI) techniques have been used to gain insight into structural and functional brain development in the first years of life [1]. Developmental changes in brain metabolite concentrations very early in life are not well understood, however. Magnetic resonance spectroscopy (MRS) is a non-invasive imaging technique used to measure *in vivo* concentrations of brain metabolites, including *N*-acetylaspartate (NAA), an index of the density of viable neurons [2]. Although MRS has been applied to the study of the neonatal brain (S1 Table), the vast majority of these studies have focused primarily on preterm or clinical samples, such as those with traumatic brain injury or hypoxia-ischemia. In addition, few studies have examined sex differences in neurochemical metabolites in the brain during early life, though animal models have revealed sex differences in a range of early neurodevelopmental processes [3], and sexual dimorphisms in brain structure and function have been found in infancy [1, 4, 5].

The vast majority of MRS studies on neonatal populations have used single-voxel spectroscopy. In comparison to single-voxel spectroscopy, multi-voxel spectroscopic techniques, such as multiplanar chemical shift imaging (MPCSI) [6–8], have many advantages, including higher spatial resolution, fewer partial volume effects because more voxels are positioned in a single tissue type, and a larger total coverage area that permits regional localization of the effects of interest [9, 10]. The main goal of this study was to use MPCSI to examine age correlates and sex differences in metabolite concentrations across the brain in healthy newborns.

### NAA, creatine, and choline

MRS studies of NAA, creatine (Cr), and choline (Cho) yield important information about neurochemical processes. NAA, present primarily in neurons [2, 11], contributes to signaling between neurons and oligodendrocytes and participates in myelin synthesis by oligodendrocytes [12]. Cr is involved in cellular energy metabolism and storage, and Cho is a marker of the structural integrity and turnover of cell membranes [2].

### Age correlates of neonatal brain metabolite concentrations

NAA concentrations during the neonatal period have been reported to increase significantly with age in both white and gray matter, and in multiple brain regions that include the thalamus, basal ganglia, hippocampus, frontal cortex, and cerebellum (S1 Table). In a study of preterm neonates, NAA and Cr concentrations in the basal ganglia, centrum semiovale, and cerebellum increased with post-conception age (from 30–43 weeks), with NAA displaying the greatest increases and no significant age correlates detected for Cho [13]. One large study of a patient radiological database reported dramatic postconceptional increases in NAA and Cr concentrations with age in several white matter and cortical and subcortical gray matter regions in the first three postnatal months [14]. No prior studies have used high-spatial-resolution, whole-brain spectroscopic imaging techniques such as MPCSI to investigate age correlates of brain metabolite concentrations in healthy newborns.

### Sex differences in neonatal brain metabolites

Animal studies have demonstrated sex differences in a variety of neurodevelopmental processes, ranging from the rate of neurogenesis to synaptic physiology [3]. Many of these differences originate prenatally, when exposure to gonadal hormones differs across fetuses [15]. Although several studies have reported sexual dimorphisms in brain structure in infancy [1, 16], sex differences in brain metabolite concentrations have not been assessed extensively in neonates or infants [13, 14]. One single-voxel MRS study of mostly preterm neonates reported no significant sex differences in NAA, Cr, or Cho in the basal ganglia, centrum semiovale, or cerebellum [13]. To date, the extent to which brain metabolite concentrations differ by sex in healthy neonates is largely unknown.

### Neonatal brain metabolites and later developmental outcomes

Higher neonatal NAA concentrations have been associated with more optimal developmental outcomes in clinical populations (S2 Table) [17–19]. In a longitudinal study of preterm neonates, steeper increases in the NAA/Cho ratio in basal ganglia nuclei and white matter regions between 28 and 40 weeks postmenstrual age (PMA) were associated with better subsequent motor, language, and cognitive performance at 18 months corrected age [20]. Other studies, however, have not found significant associations (S2 Table) [21, 22]. Collectively, metabolite research to date has been performed primarily on infants with significant clinical diagnoses that affect the brain. Given that findings from these studies may not generalize to healthy infants, research is needed on typically developing neonates. Furthermore, most previous studies have reported metabolite ratios (e.g., NAA/Cr, NAA/Cho), which are difficult to interpret from a developmental perspective, as both the numerator and denominator of a ratio can vary with age [2, 23].

Moreover, single-voxel spectroscopy has been used in the vast majority of studies examining associations between age and neonatal brain metabolite concentrations (S1 Table), even though multi-voxel spectroscopy methods, such as MPCSI, have several advantages over single-voxel spectroscopy [10]. Single-voxel spectroscopy does not simultaneously measure metabolite levels across a large number of brain regions, and thus the regional specificity of the metabolic findings in neonates (e.g., anterior vs. parietal, temporal, or subcortical) and their tissue specificity (i.e., gray matter [GM] vs. white matter [WM]) is difficult to discern [24]. Multi-voxel spectroscopy methods, on the other hand, can be used to measure metabolites in contiguous voxels across most of the brain [6] and at a higher spatial resolution than is possible using single-voxel spectroscopy [6–8].

### Current study

Many cognitive, emotional, and behavioral problems are thought to have their origins early in development, and many have prominent sex-specific differences in prevalence and symptom severity [25]; contributors to adverse health outcomes likely are represented in brain metabolite concentrations in early infancy [26–29]. Therefore, mapping the metabolic correlates of age and sex in the infant brain is important to understand the determinants of later health and illness. The goals of this study were to examine age and sex differences in NAA, Cr, and Cho levels in the healthy newborn brain and to investigate associations of neonatal brain metabolite levels with 4-month cognitive, language, and motor skills. MPCSI data were acquired approximately two weeks after birth in 41 infants (39% female; 36–42 gestational weeks at birth) of healthy adolescent or young adult mothers (14–20 years). The Bayley Scales of Infant and Toddler Development, Third Edition (Bayley-III) [30] and the mobile conjugate reinforcement paradigm, an assessment of learning and memory [31], were administered at 4 months of age. We hypothesized that postmenstrual age (PMA) at the time of MRI scanning would correlate positively with NAA concentrations across widespread regions of the brain. We also expected to detect sex differences in NAA concentrations, although the predicted direction and locations could not be specified [1]. Finally, we explored whether NAA concentrations shortly after birth were associated with developmental outcomes at 4 months of age. Cr and Cho were included as exploratory analyses that would aid interpretation of NAA findings.

## Methods

### Participants

Data used in this study came from a larger study of healthy, nulliparous pregnant adolescents and young adults who were actively engaged in prenatal care. They were recruited through the Department of Obstetrics and Gynecology at Columbia University Irving Medical Center (CUIMC) and Weill Cornell Medical College, and flyers posted in the local vicinity. Interested individuals were contacted by phone and screened for eligibility. Exclusionary criteria were smoking, use of recreational drugs, lack of fluency in English, and use of nitrates, steroids, beta blockers, triptans, or psychiatric medications.

Of the 154 mothers who enrolled in the study and were offered newborn MRI scans and infancy follow-up sessions, 82 newborns underwent MRI scans, and MPCSI data were acquired in 41 newborns. This pulse sequence was subsequently discontinued at our location following an upgrade that did not support the MPCSI pulse sequence, thus precluding scanning more infants. Mothers (93% Hispanic/Latina) were 14–20 years of age (70% were 18–19 years old). Infants (39% female) were born at 36–42 gestational weeks with typical birth weights; they ranged in PMA from 39–47 weeks at the time of MRI scan (Table 1). At 4 months, 27 and 26 infants had usable data for the Bayley-III and mobile conjugate reinforcement task, respectively. There were no major differences between those with and without developmental data at 4 months (S1 Text).

**Table 1:** Descriptive statistics for sample characteristics (*N* = 41).

	*M* (*SD*) or % (*n*)	Range
**Mother**		
** Age (years)**	17.7 (1.4)	14–20
**Race/ethnicity**		
** Hispanic/Latina**	93% (38)	
** African American**	7% (3)	
** Pre-pregnancy body mass index**	24.2 (5.5)	15.1–39.2
** Cesarean section**	15% (6)	
** Birth complications**	15% (6)	
** Infection**	2% (1)	
** Preeclampsia/hypertension**	2% (1)	
** Other**	10% (4)	
**Infant**		
** Gestational age at birth (weeks)**	39.3 (1.2)	36.4–41.6
** Postnatal age at scan (weeks)**	2.7 (1.3)	0.5–6.6
** Postmenstrual age at scan (weeks)**	42.2 (1.6)	38.7–47.0
** Postnatal age at 4-month time point (weeks)**^**a**^	18.7 (1.3)	
** Sex (female)**	39% (16)	
** Birth weight (grams)**	3193.4 (430.1)	2475.0–4315.0

^a^ *n* = 27

### Procedures

Mothers brought their infants for MRI scans as newborns and for developmental assessments at age 4 months. Infant medical records were used to ascertain gestational age at birth, birth weight, pregnancy and birth complications, and sex. Written informed consent was provided by all mothers, and all procedures were approved by the Institutional Review Board of the New York State Psychiatric Institute.

### MRI acquisition

Non-sedated infants underwent MRI scanning on a 3T whole body MR scanner (GE Health Care, Milwaukee, WI) equipped with an 8–channel head coil. MPCSI data were acquired in 6 axial oblique slices parallel to the AC-PC line, with the second bottom-most slice containing the AC-PC plane. Parameters for the MPCSI sequence were: TR = 2800 ms, TE = 144 ms, spectral width = 2000 Hz, number of complex data points = 512, FOV = 24 cm, slice thickness = 10.0 mm, slice spacing = 2.0 mm, number of phase encoding steps = 24 x 24. Water signal was suppressed using the CHESS sequence. Lipid signal was suppressed by placing eight angulated saturation bands around the brain. MPCSI data were spatially registered to a template brain using a localizer image of high in-plane resolution in the same orientation and slice locations as the MPCSI data. Those data were acquired using a 2D spin echo sequence with the following parameters: TR = 300 ms, TE = 10 ms, FOV = 24 cm, slice thickness = 10.0 mm, spacing = 2.0 mm, acquisition matrix = 256 x 128, image zero-padded to 256 x 256. We also acquired T2-weighted images for normalizing MRS data into a common template space. Parameters for the 2D spin echo sequence used to acquire the T2-weighted image were as follows: TR = 10,000 ms, TE = 129 ms, FOV = 100 cm, slice thickness = 1.0 mm, spacing = 1.0 mm, acquisition matrix = 192 x 192.

We used a long echo time (TE = 144 ms) because it flattened the spectral baseline and minimized the contamination of metabolite signals from macromolecules, thereby improving the accuracy of spectral fitting for metabolite quantification. However, the long TE limited metabolite measurement to NAA, Cr, and Cho, because these metabolites are present in greater abundance than other metabolites in the brain [32].

### MPCSI processing

Full MPCSI processing procedures are provided in the S1 Text. A brief summary of these procedures is provided here. Rigorous quality assurance procedures were employed to ensure that the data used in analyses were not corrupted by noise or participant motion. More specifically, MRS data quality was assured by reconstructing the data, assessing it for excess noise, and examining the spectrum in each voxel for baseline distortions, signal contamination by lipid signal from the scalp, incorrect placement of suppression bands, or broadening of line width. Voxels with any of these artifacts or lipid contamination were rejected and were not processed further.

**Fig 1:**
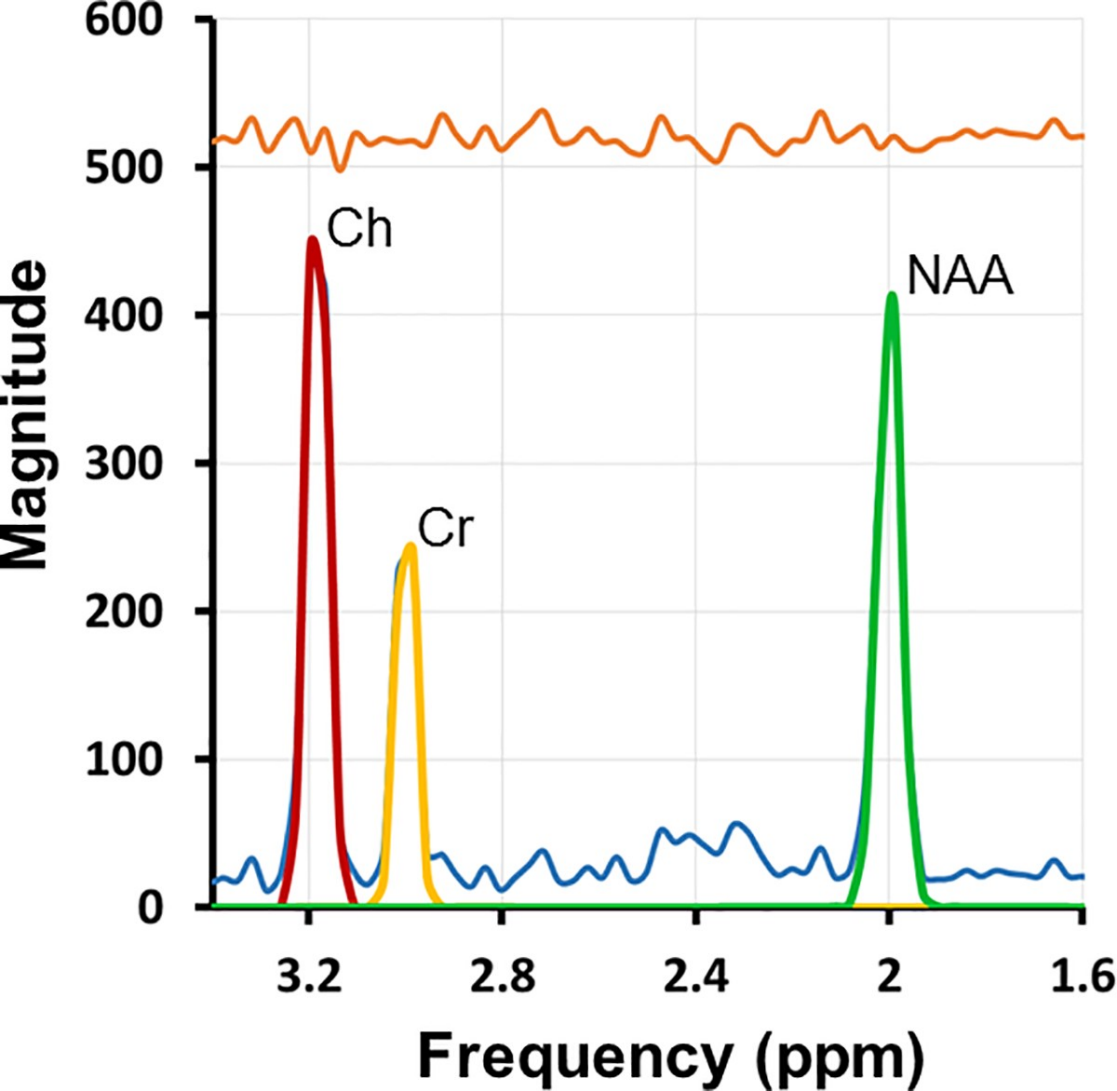
MR spectrum of representative voxel in developing white matter. Principal resonances were fit for NAA plus N-acetyl-aspartyl-glutamate (2.0 parts per million [ppm]; green), Cr plus phosphocreatine (3.0 ppm; yellow), Ch compounds (3.2 ppm; red), as well as contaminating lipids (blue). The quality of the spectra and signal-to-noise are high. The orange line at the top of the figure is the residual after fitting metabolite peaks to the MR spectrum.

We processed signal from each coil of the 8-channel head coil separately before combining their processed MRS signals to generate the spectroscopic images [33]. The combined signal was then loaded into the software *3DiCSI* (3D Interactive Chemical Shift Imaging) [34] to identify MRS voxels within the brain and save spectral data for those voxels [6]. We used model-based spectral fitting to model the spectrum in each voxel with Voigtian curves to the peaks for NAA, Cr, and Cho metabolites (S1 Text). Background noise was calculated as the standard deviation of the real part of the complex data in the regions free of signal from metabolites. A spectroscopic image for each metabolite was then generated as the ratio of the peak area to the background noise for each MRS voxel within the brain, accounting for variations in receiver and transmitter gain. Fig 1 shows a representative spectrum in a voxel of the MPCSI dataset.

We corrected the spectroscopic images for partial-volume effects and for the MPCSI point-spread function. For each MPCSI voxel and metabolite, we used linear regression to estimate the concentration of that metabolite in brain tissue and CSF using the levels of that metabolite in neighboring voxels and the proportions of brain tissue and CSF in each voxel. During spatial normalization, MPCSI data for each participant were coregistered into the coordinate space of a T2-weighted image of a template brain. The template brain was selected from our cohort of newborns using procedures that ensured the template brain was morphologically the most representative brain in the cohort (S1 Text).

The MPCSI saturation bands applied to suppress lipid signal from the scalp were not as precisely shaped as the scalp, and they unavoidably suppressed metabolite signals from several portions of cortical gray matter. Consequently, metabolite measures for many participants were available only in voxels of white matter and deep gray matter nuclei. We therefore presented results at only those voxels that had usable data from 75% or more of the infants.

### Measures

PMA at Scan PMA at the time of MRI scan is the sum of gestational age at birth and postnatal age at the time of scan [35]. Gestational age at birth was calculated based on ultrasound examinations and the date of the last reported menstrual cycle from the medical record.

Cognitive, Language, and Motor Skills The Bayley-III [30] was administered at postnatal age 4 months to assess cognitive, language, and motor skills (S1 Text). Raw and age-standardized scores (*M* = 100, *SD* = 15) were calculated; raw scores were used in analyses. The Bayley-III has acceptable psychometric properties and norms based on a racially- and socioeconomically-representative sample [30, 36], although problems with Bayley-III reliability and validity have been noted [37–39].

Learning and Memory At 4-months postpartum, mothers brought their infants to the lab on two consecutive days for participation in the mobile conjugate reinforcement paradigm [31, 40] (S1 Text). After placing infants in a specially set up crib, each videotaped 15-min session began with a 3-min non-reinforcement phase (baseline), followed by a 9-min reinforcement phase (three 3-min learning blocks), and a final 3-min non-reinforcement phase (immediate retention). During periods of reinforcement, when infants kicked, a mobile hanging above the crib bounced, whereas during periods of non-reinforcement, the mobile did not bounce as a result of their kicking. On Day 1, the initial 3-min period of non-reinforcement (baseline) provides a measure of the infant's baseline kick rate, and the final 3-min period of non-reinforcement provides a measure of the infant's immediate retention. On Day 2, kicking during the initial period of non-reinforcement (Day 2 baseline) reflects the infant's long-term (24-hr) retention of the contingency.

Trained coders used the videotapes to count the number of times infants kicked per minute. A second trained coder independently coded 20% of the sessions. Inter-rater reliability was high (Spearman rank correlation  =  0.95; range: 0.88–0.99). Ratio scores were calculated by dividing the mean kick rate during each learning and retention block by the mean kick rate during Day 1 baseline [40].

### Statistical analyses

Descriptive statistics were computed using SAS (version 9.4). Multiple linear regression was applied voxel-wise throughout the brain, using an in-house developed program, separately for each of the three metabolite values (NAA, Cho, and Cr) entered as the dependent variable and with PMA at scan and sex entered simultaneously as independent variables. Similarly, NAA, Cho, and Cr concentrations were regressed voxel-wise onto Bayley-III and mobile conjugate reinforcement paradigm scores, with PMA and sex included as covariates.

We used the Benjamini–Yekutieli procedure [41] for False Discovery Rate (FDR) to control for false positives within each statistical map [42–44]; *p*-values surviving FDR correction at a corrected *p* < 0.05 level were color-coded as statistical parametric maps on the T2-template. This FDR procedure is a rigorous statistical procedure that limits false discovery in findings at a specified rate, which in our analyses is 0.05. This procedure will ensure that no more than 5% of the reported findings are generated by chance due to noise in the data. By permitting a prespecified fraction of false positives in our findings, this procedure provides significantly greater statistical power to detect true findings in the data. Furthermore, FDR is adaptive and adjusts the threshold for significant findings depending on the noise in the data. In contrast, other alternative familywise error rate procedures, such as Holm-Bonferroni correction, control for false positives across the entire family of hypotheses tested at a specified significance level α. Although Bonferroni correction will ensure that the probability of finding one or more false positive is smaller than α across all hypotheses, this procedure is highly conservative [45] providing low statistical power and therefore too often failing to detect true positives. Thus we opted to balance false positive and false negative findings through use of FDR correction.

## Results

Descriptive statistics for the Bayley-III and mobile conjugate reinforcement paradigm are presented in Table 2. The infants in this study demonstrated typical patterns of learning and memory performance on the mobile conjugate reinforcement paradigm (see also [40]).

**Table 2:** Descriptive statistics for 4-month developmental outcomes.

Bayley-III scale	*N*	*M*	*SD*
Cognitive raw score	27	16.78	4.31
** **Standard score	27	95.37	16.69
Language raw score	27	20.30	4.09
** **Standard score	27	101.15	11.93
Motor raw score	27	19.52	5.76
** **Standard score	27	94.48	23.69
**Mobile conjugate reinforcement paradigm**	***N***	***M***	***SD***
Learning block 1 ratio score	25	2.69	2.93
Learning block 2 ratio score	22	3.29	2.99
Learning block 3 ratio score	20	4.69	6.01
Immediate retention ratio score	18	4.57	5.83
Long-term retention ratio score	25	1.58	1.34

### Postmenstrual age correlates positively with newborn brain metabolite concentrations

**Fig 2:**
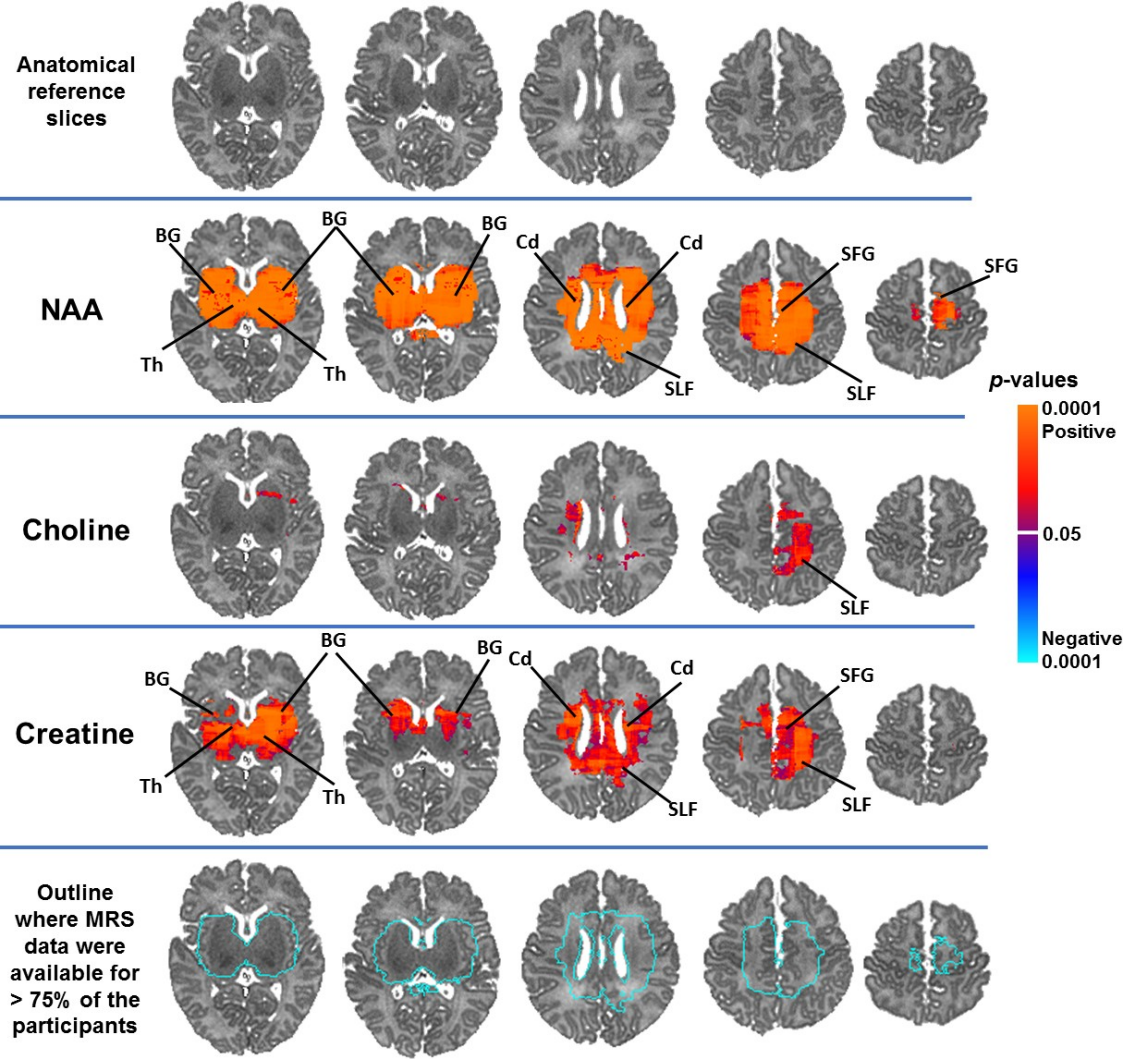
NAA concentrations significantly and positively correlated with PMA at scan in widespread white and subcortical gray matter regions in healthy newborns, controlling for sex. Findings for creatine and choline were weaker but in the same direction and in overlapping brain regions as the findings for NAA. Anatomical reference slices are presented in the top row. Maps indicating the voxels for which at least 75% of the sample had usable data are presented in the bottom row. FDR correction for multiple comparisons was done with FDR at *p* < .05. Warm colors (red, orange) indicate significant positive associations and cool colors (shades of blue) indicate significant inverse associations, with higher degrees of warmth/coolness corresponding to smaller FDR-corrected *p*-values as shown in the color bar. SLF, superior longitudinal fasciculus; BG, basal ganglia; Th, thalamus; SFG, superior frontal gyrus; Cd, caudate.

**Fig 3:**
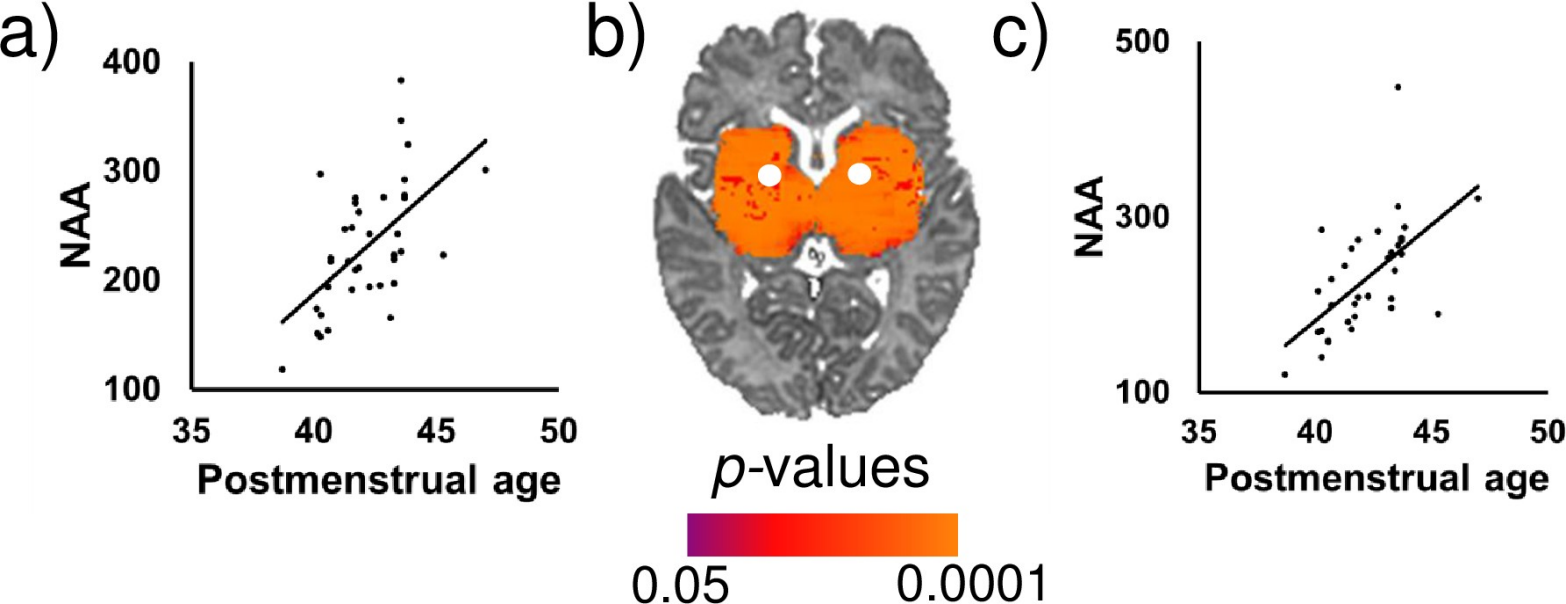
Postmenstrual age at scan (in weeks) was significantly positively associated with NAA concentrations in the (a) left and (c) right basal ganglia after controlling for sex. FDR-corrected *p*-values are shown in the color bar. Metabolite data were sampled from the regions indicated by the white dots (b). NAA is shown in arbitrary units (a.u.).

**Fig 4:**
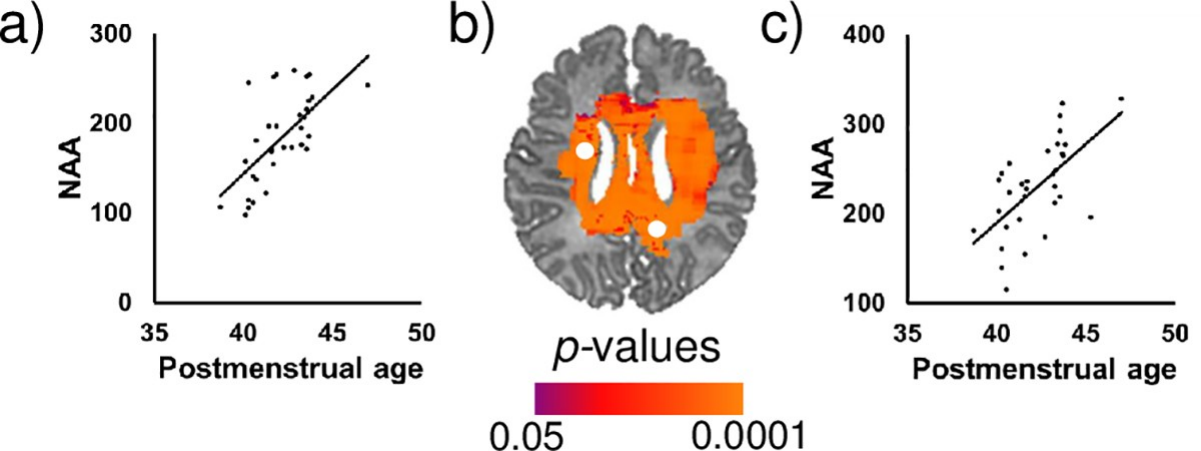
Postmenstrual age at scan (in weeks) was significantly positively associated with NAA concentrations in the (a) left caudate and (c) right superior longitudinal fasciculus after controlling for sex. FDR-corrected p-values are shown in the color bar. Metabolite data in the scatterplots were sampled from the regions indicated by the white dots (b). NAA is shown in arbitrary units (a.u.).

Controlling for infant sex, advancing PMA at scan was significantly associated with higher NAA concentrations in white matter (superior longitudinal fasciculus), superior frontal gyrus, and subcortical gray matter (thalamus, basal ganglia) (Fig 2). Similar results were evident for Cr and, to a lesser extent, Cho. Representative scatterplots are presented in Figs 3 and 4. Effect sizes for PMA at scan across voxels are presented in S1 Fig.

### Female newborns have higher brain metabolite concentrations than male newborns

**Fig 5:**
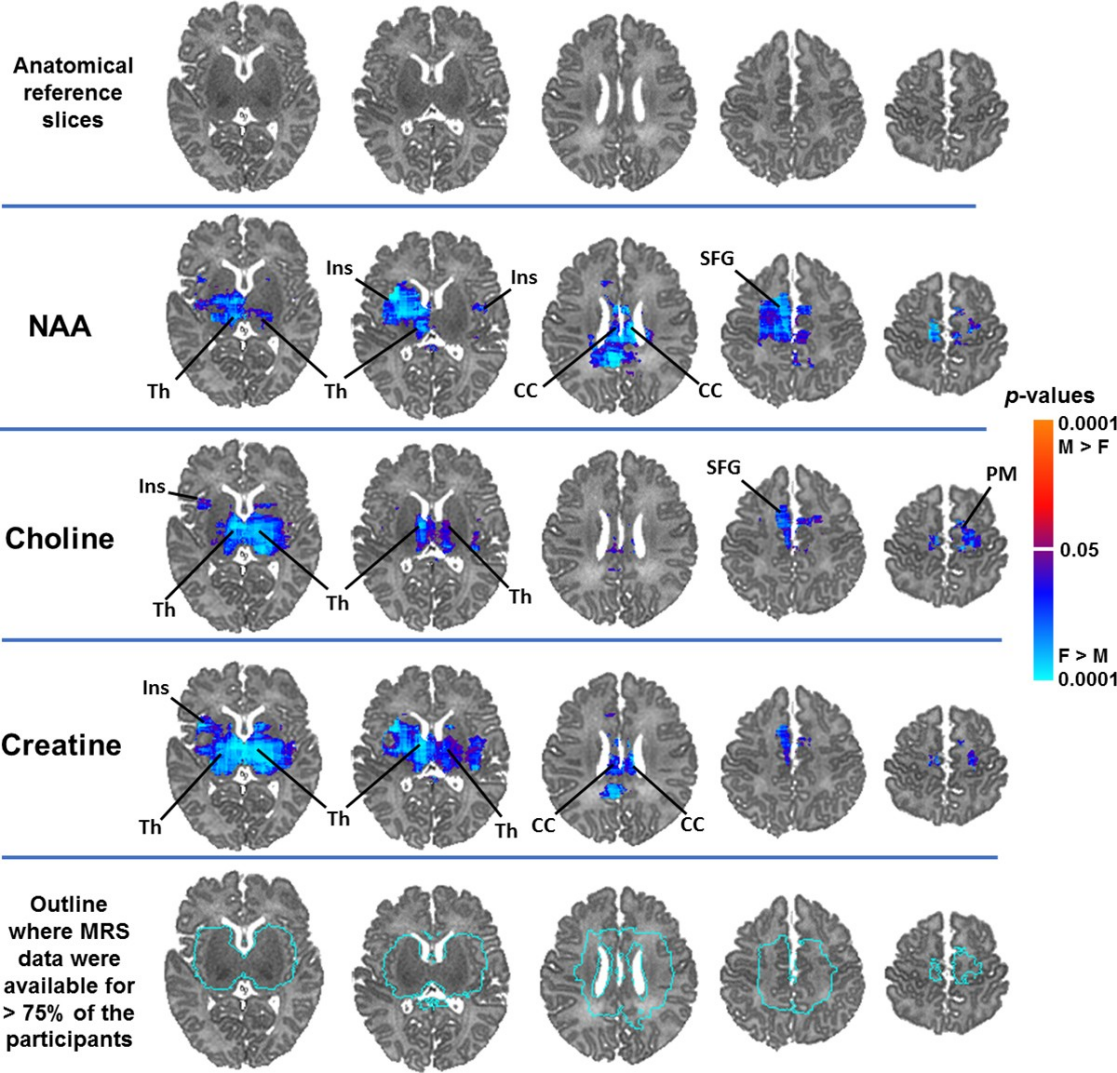
Newborn females had higher NAA, creatine, and choline concentrations in frontal white matter and subcortical gray matter regions compared to newborn males, controlling for PMA at scan. Anatomical reference slices are presented in the top row. Maps indicating the voxels for which at least 75% of the sample had usable data are presented in the bottom row. FDR correction for multiple comparisons was done with FDR at *p* < .05. Cool colors (shades of blue) indicate significantly higher metabolite concentrations in females compared to males. The magnitude of FDR-corrected *p*-values is color-coded as shown in the color bar. Th, thalamus; SFG, superior frontal gyrus; Ins, insula; PM, premotor cortex; CC, corpus callosum.

**Fig 6:**
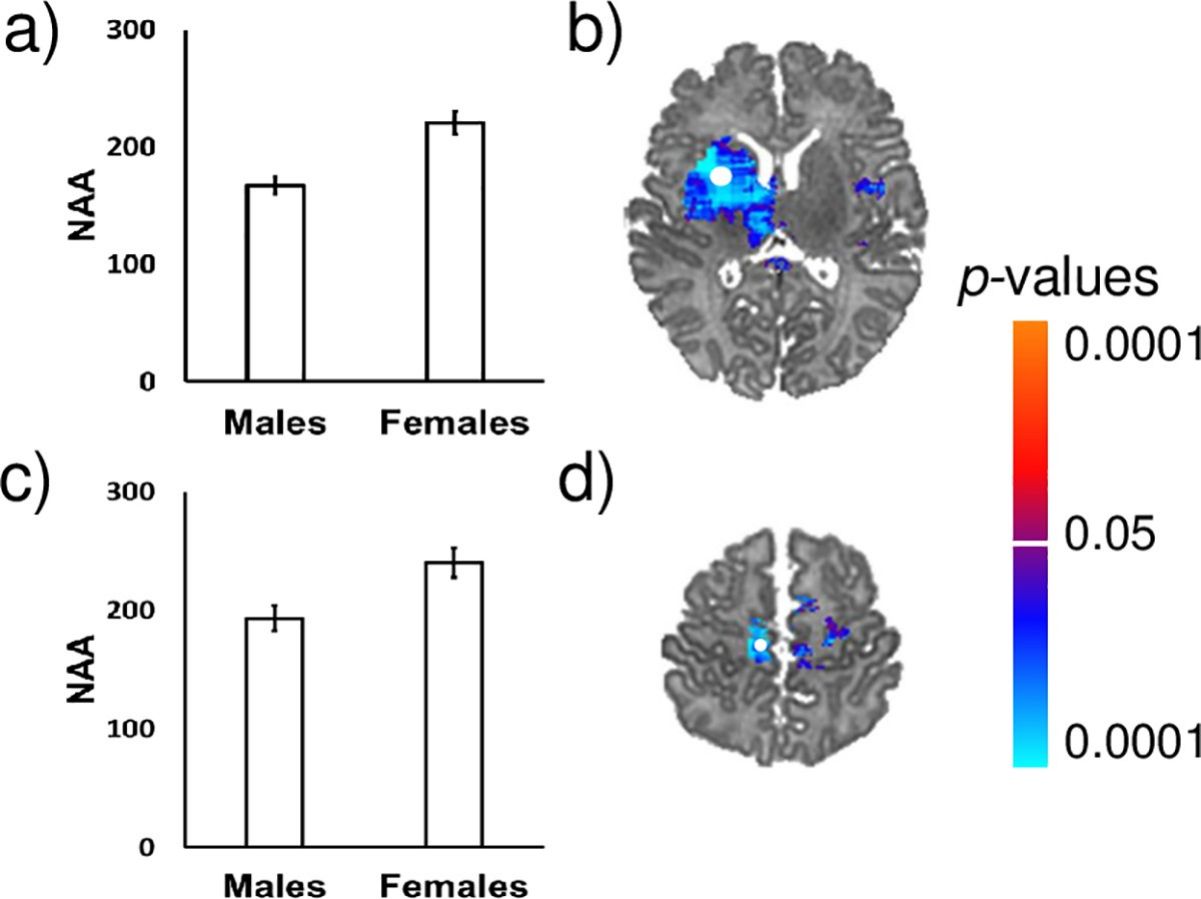
Bar graphs showing sex differences in NAA concentrations in the healthy newborn brain. Females had higher NAA concentrations than males in the (a) insula and (c) superior frontal gyrus. FDR-corrected p-values are shown in the color bar. The error bars represent standard error (SE). Metabolite data in the scatterplots were sampled from the regions indicated by the white dots (b and d). NAA is shown in arbitrary units (a.u.).

Females compared to males had greater NAA concentrations in white matter (corpus callosum), subcortical gray matter (thalamus), insula, and superior frontal gyrus. Females compared to males had greater Cho concentrations in the thalamus, insula, superior frontal gyrus, and premotor regions. Females compared to males had greater Cr concentrations in the thalamus, corpus callosum, and insula (Fig 5). Bar graphs for representative voxels are presented in Fig 6. Effect sizes for sex across voxels are presented in S2 Fig.

### Newborn NAA concentrations correlate with 4-month learning and memory

**Fig 7:**
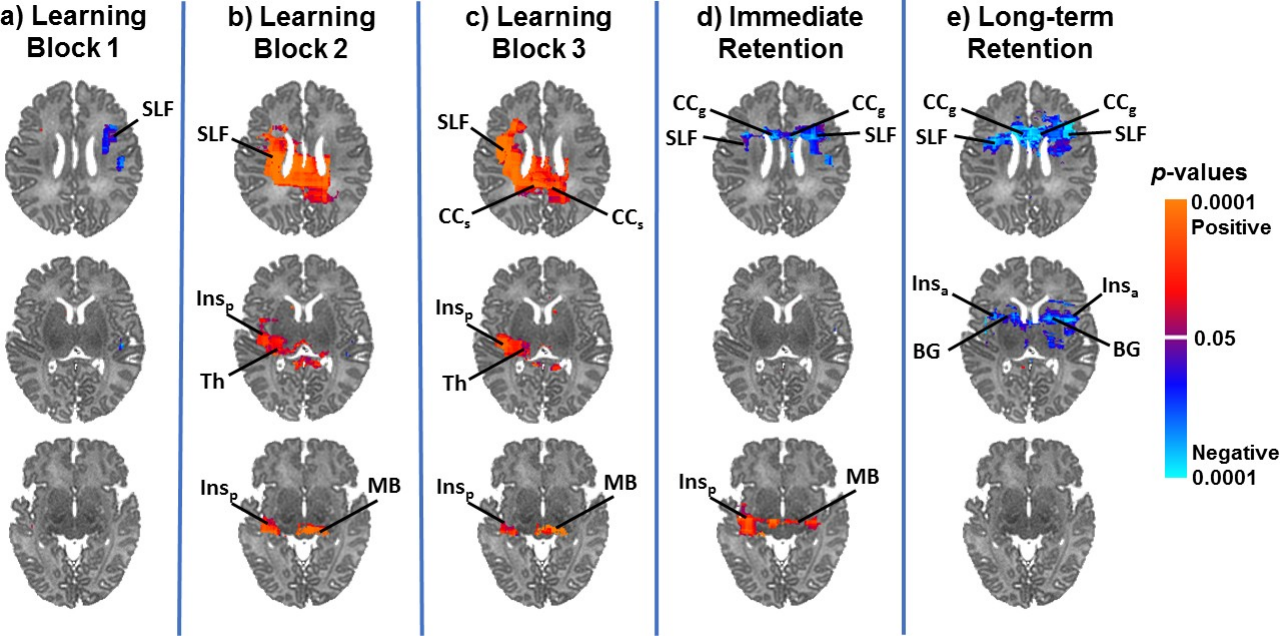
Neonatal NAA concentrations in white matter and subcortical gray matter regions were significantly associated with performance on the mobile conjugate reinforcement paradigm at 4 months of age, controlling for PMA and sex. NAA concentrations in the SLF and splenium of the corpus callosum were significantly positively associated with learning (b and c). NAA concentrations in the SLF and genu of the corpus callosum were significantly inversely associated with immediate and long-term retention (d and e). P-values are FDR-corrected and color coded if corrected *p* is < .05. Warm colors (red, orange) indicate significant positive associations and cool colors (shades of blue) indicate significant inverse associations, with higher degrees of warmth/coolness corresponding to smaller FDR-corrected *p*-values as shown in the color bar. SLF, superior longitudinal fasciculus; CCs, splenium of corpus callosum; CCg, genu of corpus callosum; Ins_p_, posterior insula; Th, thalamus; Ins_a_, anterior insula; BG, basal ganglia; MB, midbrain.

**Fig 8:**
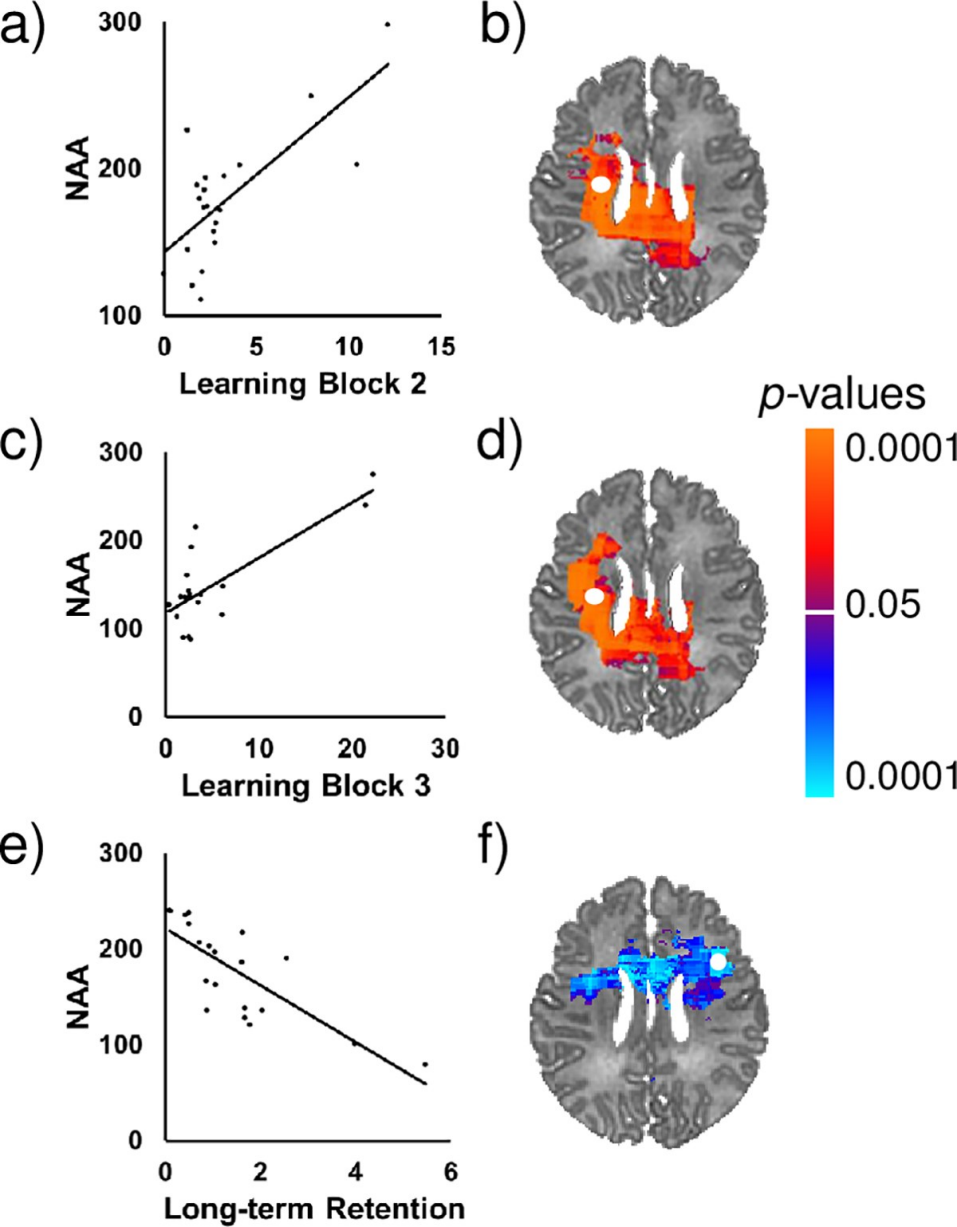
Scatterplots for representative voxels showing correlation of neonatal NAA concentrations with 4-month performance on the mobile conjugate reinforcement paradigm. NAA concentrations in the SLF (b and d) were significantly positively associated with learning skills (a and c), while controlling for PMA and sex. NAA concentrations in a different portion of the SLF (f) were significantly inversely associated with long-term retention (e). FDR-corrected p-values are shown in the color bar. Metabolite data in the scatterplots were sampled from the regions indicated by the white dots. NAA is shown in arbitrary units (a.u.).

Neonatal NAA concentrations correlated significantly with performance on the mobile conjugate reinforcement paradigm at 4 months of age. Higher NAA concentrations in the superior longitudinal fasciculus (SLF), corpus callosum, insula, and thalamus were associated with greater learning. In contrast, lower NAA concentrations in the corpus callosum, SLF, insula, and basal ganglia were associated with greater immediate and long-term retention (Fig 7). Scatterplots for representative voxels are presented in Fig 8. Effect sizes for scores on this task across voxels are presented in S3 Fig. Newborn brain metabolite concentrations were not significantly associated with performance on the Bayley-III at 4 months.

## Discussion

The goals of this study were to examine postmenstrual age (PMA)- and sex-related variability in brain metabolite concentrations in healthy neonates and the associations of neonatal brain metabolite levels with 4-month developmental outcomes. Previous MRS studies with infants have focused primarily on preterm or clinical samples and have not examined sex differences. In addition, previous research has largely used single-voxel spectroscopy techniques (S1 Table), whereas multi-voxel spectroscopic techniques, such as MPCSI, have higher spatial resolution and can shed light on regional specificity of the associations [6–8]. This study is the first to use MPCSI to assess concentrations of NAA, Cr, and Cho in the healthy newborn brain.

### Age correlates of neonatal NAA concentrations

As hypothesized, older PMA was significantly associated with higher NAA concentrations in anterior and posterior deep white matter and subcortical gray matter. NAA is synthesized in the mitochondria of neurons and stored largely in neuronal bodies and their axons. A portion of its signal may be attributable to N-acetyl-aspartyl-glutamate (NAAG), a prominent neuropeptide that regulates glutamate and dopamine release [46, 47]. Increasing NAA concentrations reflect greater mitochondrial energy metabolism, demands for lipid synthesis, or NAAG [12, 48]. Our findings of strong positive correlations of NAA concentrations with increasing PMA suggest rapid developmental increases in the density of neuronal tissue in these regions in early postnatal life [2].

PMA-related increases in NAA were found in the basal ganglia and thalamus, which undergo rapid morphological growth in early life [1, 49]. Sensorimotor portions of the basal ganglia, together with the sensorimotor cortex, drive motor learning and control. The thalamus relays basal ganglia output to the cortex and mediates information flow between cortical circuits. PMA-related increases in NAA in these structures during the perinatal period are consistent with the rapid rates of synaptogenesis and dendritic growth that characterize this developmental period in these nuclei (Gilmore et al., 2018).

PMA-related increases in NAA were also found in higher-order association areas and associative fiber bundles (e.g., frontal white matter, SLF). The SLF, which connects dorsal frontal with inferior and superior parietal cortices, is a component of neural networks that subserve numerous cognitive functions, including attention and self-regulation [50]. Increases in NAA concentrations may contribute to the rapid early developmental increases in organization (fractional anisotropy [FA]) of white matter tracts, including the SLF [1, 6, 51]. PMA-related increases in NAA in white matter are consistent with rapid myelination during the first year of life [1, 52, 53] and the lipid synthesis it demands [12, 54].

### Sex differences in neonatal NAA concentrations

Females compared to males had higher NAA concentrations in cerebral white matter (insula, superior frontal gyrus, corpus callosum), premotor cortex, and subcortical gray matter (thalamus). These findings are consistent with an emerging literature showing infant sex differences in regional gray and white matter volumes [1, 4, 5, 49, 55] and white matter microstructure [1, 56, 57], and with previous theoretical and empirical work suggesting that the brain may develop earlier and more rapidly in females than in males [16, 58, 59]. For example, female newborns have a higher rate of myelination in the corpus callosum between 3 and 60 months [56], higher FA values across widespread white matter regions [57], and larger volume of the motor cortex [4].

Given that the sex differences we found were present shortly after birth, they are likely the product of hormonal and genetic influences on the fetal and newborn brain, rather than of differential environments and experiences in postnatal life [3, 60]. Differential exposure to gonadal steroid hormones and differential spatial and temporal profiles of receptors for those hormones in male and female fetuses produces sexual dimorphisms in brain structure and function [15, 61]. It has been suggested that testosterone may slow neural development, and some animal research has supported this conjecture [58]. These sex differences in prenatal brain development may lead to sex differences in how infants elicit and respond to stimulation from their environment during postnatal life [62–65].

### Associations of neonatal NAA concentrations with 4-month developmental outcomes

Newborn NAA concentrations were significantly associated with 4-month developmental outcomes as measured by the mobile conjugate reinforcement paradigm. Greater NAA concentrations in the SLF and corpus callosum were associated with higher learning scores on the task. These findings are consistent with previous work in preterm samples that has associated higher neonatal NAA levels with higher scores on developmental measures [17–19, 66], although other studies have not found these associations (S2 Table) [21, 22, 67]. In contrast, *lower* NAA concentrations in these locations were associated with higher retention scores on the task. It is possible that these lower levels may signify a slower rate of development or more prolonged maturation that supports greater memory task performance, consistent with previous longitudinal MRI work on the neural underpinnings of general cognitive development [68].

### Age correlates of neonatal creatine and choline concentrations

Cr concentrations also increased with advancing PMA at scan, albeit less dramatically and in fewer brain regions compared with NAA. Findings for Cho were similar but even more attenuated. This pattern of findings across metabolites is consistent with previous studies in which age correlates for NAA and Cr in neonates have been detected more frequently than for Cho (S1 Table) [13, 14, 69, 70]. Age-related increases in Cr may represent an increase in energy metabolism or the need for increased energy reserve with advancing age, whereas increases in Cho likely reflect an increased rate of cell membrane synthesis or turnover. The common pattern of findings, in which age correlates were in the same direction and overlapping regions may also be driven by a developmental increase in mass and density of neuronal tissue, which will necessarily entail an increase in all metabolites that support neuronal processes, including cell energetics and membrane turnover.

### Limitations

This study had several limitations. Interpretation of age correlates from cross-sectional data should be regarded with caution [71], as definitive identification of developmental trajectories requires repeated measures over time. Metabolite concentrations were not available for most cortical gray matter over the surface convexities, because MPCSI saturation bands applied to suppress lipid signal from the scalp were not as precisely shaped as the scalp, and they therefore unavoidably suppressed metabolite signals from cortical gray matter. Attrition in follow-up also limited our sample size and statistical power for testing correlations of metabolite concentrations with 4-month developmental outcomes. In addition, all infants were born to adolescent or young adult mothers, who are often considered an at-risk population, though screening and exclusionary criteria ensured a healthy, reasonably representative sample of newborns for women in this age group. Nonetheless, findings from this study may not be generalizable to the children of older mothers. In addition, participant motion during image acquisition is a common concern in neuroimaging studies and could have affected the results reported here. However, the infants in this study were asleep throughout the acquisition of the MPCSI data, and their heads were immobilized. These acquisition procedures coupled with the rigorous quality assurance procedures employed in this study make it unlikely that participant motion during image acquisition corrupted the analyses or results of this study.

Results from this study shed light on typical patterns of early metabolic development across the brain, providing the basis for identifying regional developmental deviations that may signal risk for neurodevelopmental and psychiatric disorders. Sex differences in early metabolite concentrations, with females tending to have higher regional concentrations than males, may relate to the well-documented sex differences in risk for neurodevelopmental and psychiatric disorders.

## Supporting information

S1 Table Studies that investigated associations of age with neonatal brain N-acetylaspartate (NAA), creatine, and choline concentrations. (DOCX)

S2 Table Studies that investigated associations between neonatal brain metabolite concentrations and later developmental outcomes. (DOCX)

S1 Fig Strength of associations between postmenstrual age (PMA) at scan and brain metabolite concentrations. The magnitude of association is displayed in terms of beta values from multiple linear regression applied voxel-wise throughout the brain separately for each of the three metabolite values (NAA, Ch, and Cr) entered as the dependent variable and PMA at scan and sex entered simultaneously as independent variables. Anatomical reference slices are presented in the top row. Maps indicating the voxels for which at least 75% of the sample had usable data are presented in the bottom row.
(TIF)

S2 Fig The magnitude of sex differences in brain metabolite concentrations in neonates. The magnitude of the association is displayed in terms of beta values from multiple linear regression applied voxel-wise throughout the brain separately for each of the three metabolite values (NAA, Ch, and Cr) entered as the dependent variable and PMA at scan and sex entered simultaneously as independent variables. Anatomical reference slices are presented in the top row. Maps indicating the voxels for which at least 75% of the sample had usable data are presented in the bottom row.
(TIF)

S3 Fig The magnitude of associations between neonatal NAA concentrations and performance on the mobile conjugate reinforcement paradigm at 4 months. Beta values are displayed from multiple linear regression applied voxel-wise throughout the brain for NAA entered as the dependent variable and ratio score as the independent variable, with PMA and sex included as covariates. Brain maps are presented for ratio scores on learning block 2, learning block 3, and the long-term retention block (Day 2 baseline) for the mobile conjugate reinforcement paradigm.
(TIF)

S1 Text Supporting information about the methods of the study. (DOCX)
