# Supplementary material for: Neonatal brain metabolite concentrations: Associations with age, sex, and developmental outcomes
Source: PLoS One. 2020 Dec 17;15(12):e0243255. doi: 10.1371/journal.pone.0243255 (PMC7746171; doi:10.1371/journal.pone.0243255)
Supplement: S1 Table — (DOCX) [file pone.0243255.s001.docx]

**S1 Table. Studies examining associations of age with neonatal brain N-acetylaspartate (NAA), creatine, and choline concentrations**

| **Authors** | ***N*** | **Population** | **Age Range** | **Age Measure** | **MRS technique** | **Brain regions** | **Correlations with age** |
| --- | --- | --- | --- | --- | --- | --- | --- |
| Augustine et al. (2008) | 36 | Very LBW, preterm | 35-43 weeks | PMA | Multivoxel MRS (PRESS) | Thalamus, basal ganglia | Increases in NAA/choline |
| Bapat et al. (2014) | 43 | Extremely LBW and healthy term controls | 38 weeks PMA or before discharge | PMA | Single-voxel MRS (PRESS) | Subventricular zone, hippocampus, frontal cortex | Correlated with NAA/choline per prelim analyses; no other information provided |
| Basu et al. (2019) | 53 | Preterm, LBW (born ≤ 32 weeks) | 27-37 weeks PMA | PMA | Single-voxel MRS (PRESS) | Cerebellum | Increases in NAA, creatine, choline |
| Blüml et al. (2013) | 309 total across ages | Normative, all born at term (40+ gestational weeks) | 0-3 months postnatal age | PCA | Single-voxel MRS (PRESS) | Parietal white matter, frontal white matter, parieto-occipital cerebral cortex, deep gray nuclei (combined thalamus/ caudate/  putamen), ventral pons | Increases in NAA and creatine in frontal and parietal white matter, parieto-occipital gray matter, and ventral pons;  increases in NAA in deep gray nuclei |
| Brossard et al. (2017) | 59 | Preterm (born < 32 weeks) | 38-45 weeks | GA | Single-voxel MRS (PRESS) | Cerebellum | Increases in NAA, choline, creatine |
| Card et al. (2013) | 99 | Preterm (born ≤ 32 weeks GA) | 29.1-31.6 weeks PMA; another at 40.2-43.1 weeks PMA | PMA | Single-voxel MRS (PRESS) | Basal ganglia | Increases in NAA/choline and creatine/choline within both the preterm and term-equivalent age periods |
| Chau et al. (2013) | 177 | Preterm (born 24-32 weeks) | One at 27-46 weeks; another at 32-46 weeks | GA | Multivoxel chemical shift imaging | Centrum semiovale, basal ganglia | Increases in NAA/choline |
| Gadin et al. (2012) | 29 | Preterm (born < 30 weeks) and term (born ≥ 37 weeks) | 36 weeks PMA | -- | Single-voxel MRS (PRESS) | Periventricular parietal region | -- |
| Hart et al. (2014) | 67 | Preterm; born < 35 weeks | 37-44 weeks corrected GA | GA | Single-voxel MRS (PRESS) | Anterior and posterior periventricular white matter | Increases in NAA/creatine, NAA/choline, NAA |
| Hyodo et al. (2018) | 33 | Preterm (born < 37 weeks) and very LBW | 37-42 weeks PMA | GA at birth | Single-voxel (PRESS) | Bilateral frontal white matter and thalamus | Increases in NAA/choline in left frontal white matter and right and left thalamus |
| Kendall et al. (2014) | 43 | Preterm; born < 32 weeks | 37–44  weeks PMA | PMA | Single-voxel MRS (PRESS) | Left posterior periventricular white matter | No significant associations |
| Kreis et al. (2002) | 21 | Preterm and full-term; born 32-43 weeks | 32-43 weeks GA | GA | Single-voxel MRS (PRESS) | Centrum semiovale, thalamus, occipital gray matter | Increases in NAA and creatine |
| Lally et al. (2019) | 223 | Neonatal encephalopathy | 4–14 days after birth | GA at birth, postnatal age at scan | Single-voxel MRS | Thalamus | NAA concentration increased with GA at birth; no relation with postnatal age |
| Roelants-van Rijn et al. (2004) | 40 | Preterm; some small for GA | One at 32 weeks; another at 41 weeks | PMA | Single-voxel MRS (PRESS) | Basal ganglia, periventricular white matter | Increases in NAA/choline |
| Tanifuji et al. (2017) | 20 | Preterm | One at 37-46 weeks PMA; another at 64-73 weeks PMA | PMA | Single-voxel MRS (PRESS) | Right basal ganglia | Increases in NAA/creatine, NAA/choline; decrease in choline/creatine; increases in NAA and creatine but no significant difference in choline concentrations |
| Taylor et al. (2018) | 45 | Preterm (born < 32 weeks GA) | At birth (25-32 weeks) and at term-equivalent age (36-45 weeks) | PMA | Single-voxel MRS (PRESS) | Left basal ganglia | Increases in creatine/choline, NAA/choline, NAA/creatine |
| Tomiyasu et al. (2013) | 79 | 60 preterm (born 23-36 weeks); 19 term (born 37-41 weeks); all had normal radiological and clinical evaluations | 30-43 weeks PCA | PCA | Single-voxel MRS (PRESS) | Basal ganglia, centrum semiovale,  cerebellum | Increases in NAA and creatine but not choline |
| Van Kooij et al. (2012) | 56 | Preterm | 39-45 weeks PMA | PMA | Single-voxel MRS (PRESS) | Cerebellum | No significant association for NAA/choline |
| Xu et al. (2011) | 55 | Preterm; M = 28.4 weeks PCA | 30-42 weeks GA | GA | Multivoxel 3D MRS (PRESS) | Thalamus, basal ganglia, ventral (temporal) visual association  tract, calcarine gray matter, corticospinal tract, parietal white matter, frontal white matter | Increases in NAA/choline in all regions |

*Note*. PRESS, point-resolved spectroscopy; PMA, postmenstrual age; PCA, postconceptional age; GA, gestational age; LBW, low birth weight

**References**

Augustine, E. M., Spielman, D. M., Barnes, P. D., Sutcliffe, T. L., Dermon, J. D., Mirmiran, M., Clayton, D. B., & Ariagno, R. L. (2008). Can magnetic resonance spectroscopy predict neurodevelopmental outcome in very low birth weight preterm infants? *Journal of Perinatology*, *28*(9), 611–618. https://doi.org/10.1038/jp.2008.66

Bapat, R., Narayana, P. A., Zhou, Y., & Parikh, N. A. (2014). Magnetic Resonance Spectroscopy at Term-Equivalent Age in Extremely Preterm Infants: Association With Cognitive and Language Development. *Pediatric Neurology*, *51*(1), 53–59. https://doi.org/10.1016/j.pediatrneurol.2014.03.011

Basu, S. K., Pradhan, S., Kapse, K., McCarter, R., Murnick, J., Chang, T., & Limperopoulos, C. (2019). Third Trimester Cerebellar Metabolite Concentrations are Decreased in Very Premature Infants with Structural Brain Injury. *Scientific Reports*, *9*. https://doi.org/10.1038/s41598-018-37203-4

Blüml, S., Wisnowski, J. L., Nelson, M. D., Paquette, L., Gilles, F. H., Kinney, H. C., & Panigrahy, A. (2013). Metabolic Maturation of the Human Brain From Birth Through Adolescence: Insights From In Vivo Magnetic Resonance Spectroscopy. *Cerebral Cortex*, *23*(12), 2944–2955. https://doi.org/10.1093/cercor/bhs283

Brossard-Racine, M., Murnick, J., Bouyssi-Kobar, M., Coulombe, J., Chang, T., & Limperopoulos, C. (2017). Altered Cerebellar Biochemical Profiles in Infants Born Prematurely. *Scientific Reports*, *7*. https://doi.org/10.1038/s41598-017-08195-4

Card, D., Nossin-Manor, R., Moore, A. M., Raybaud, C., Sled, J. G., & Taylor, M. J. (2013). Brain metabolite concentrations are associated with illness severity scores and white matter abnormalities in very preterm infants. *Pediatric Research*, *74*(1), 75–81. https://doi.org/10.1038/pr.2013.62

Chau, V., Synnes, A., Grunau, R. E., Poskitt, K. J., Brant, R., & Miller, S. P. (2013). Abnormal brain maturation in preterm neonates associated with adverse developmental outcomes. *Neurology*, *81*(24), 2082–2089. https://doi.org/10.1212/01.wnl.0000437298.43688.b9

Gadin, E., Lobo, M., Paul, D. A., Sem, K., Steiner, K. V., Mackley, A., Anzilotti, K., & Galloway, C. (2012). Volumetric MRI and MRS and early motor development of infants born preterm. *Pediatric Physical Therapy*, *24*(1), 38–44. https://doi.org/10.1097/PEP.0b013e31823e069d

Hart, A. R., Smith, M. F., Whitby, E. H., Alladi, S., Wilkinson, S., Paley, M. N., & Griffiths, P. D. (2014). Diffusion-weighted imaging and magnetic resonance proton spectroscopy following preterm birth. *Clinical Radiology*, *69*(8), 870–879. https://doi.org/10.1016/j.crad.2014.04.001

Hyodo, R., Sato, Y., Ito, M., Sugiyama, Y., Ogawa, C., Kawai, H., Nakane, T., Saito, A., Hirakawa, A., Kidokoro, H., Natsume, J., & Hayakawa, M. (2018). Magnetic resonance spectroscopy in preterm infants: Association with neurodevelopmental outcomes. *Archives of Disease in Childhood. Fetal and Neonatal Edition*, *103*(3), F238–F244. https://doi.org/10.1136/archdischild-2016-311403

Kendall, G. S., Melbourne, A., Johnson, S., Price, D., Bainbridge, A., Gunny, R., Huertas-Ceballos, A., Cady, E. B., Ourselin, S., Marlow, N., & Robertson, N. J. (2014). White matter NAA/Cho and Cho/Cr ratios at MR spectroscopy are predictive of motor outcome in preterm infants. *Radiology*, *271*(1), 230–238. https://doi.org/10.1148/radiol.13122679

Kreis, R., Hofmann, L., Kuhlmann, B., Boesch, C., Bossi, E., & Hüppi, P. S. (2002). Brain metabolite composition during early human brain development as measured by quantitative in vivo 1H magnetic resonance spectroscopy. *Magnetic Resonance in Medicine*, *48*(6), 949–958. https://doi.org/10.1002/mrm.10304

Lally, P. J., Montaldo, P., Oliveira, V., Soe, A., Swamy, R., Bassett, P., Mendoza, J., Atreja, G., Kariholu, U., Pattnayak, S., Sashikumar, P., Harizaj, H., Mitchell, M., Ganesh, V., Harigopal, S., Dixon, J., English, P., Clarke, P., Muthukumar, P., … Thayyil, S. (2019). Magnetic resonance spectroscopy assessment of brain injury after moderate hypothermia in neonatal encephalopathy: A prospective multicentre cohort study. *The Lancet. Neurology*, *18*(1), 35–45. https://doi.org/10.1016/S1474-4422(18)30325-9

Roelants-van Rijn, A. M., van der Grond, J., Stigter, R. H., de Vries, L. S., & Groenendaal, F. (2004). Cerebral structure and metabolism and long-term outcome in small-for-gestational-age preterm neonates. *Pediatric Research*, *56*(2), 285–290. https://doi.org/10.1203/01.PDR.0000132751.09067.3F

Tanifuji, S., Akasaka, M., Kamei, A., Araya, N., Asami, M., Matsumoto, A., Sotodate, G., Konishi, Y., Shirasawa, S., Toya, Y., Kusano, S., Chida, S., Sasaki, M., & Matsuda, T. (2017). Temporal brain metabolite changes in preterm infants with normal development. *Brain & Development*, *39*(3), 196–202. https://doi.org/10.1016/j.braindev.2016.10.006

Taylor, M. J., Vandewouw, M. M., Young, J. M., Card, D., Sled, J. G., Shroff, M. M., & Raybaud, C. (2018). Magnetic resonance spectroscopy in very preterm-born children at 4 years of age: Developmental course from birth and outcomes. *Neuroradiology*, *60*(10), 1063–1073. https://doi.org/10.1007/s00234-018-2064-7

Tomiyasu, M., Aida, N., Endo, M., Shibasaki, J., Nozawa, K., Shimizu, E., Tsuji, H., & Obata, T. (2013). Neonatal Brain Metabolite Concentrations: An In Vivo Magnetic Resonance Spectroscopy Study with a Clinical MR System at 3 Tesla. *PLoS ONE*, *8*(11). https://doi.org/10.1371/journal.pone.0082746

Van Kooij, B. J. M., Benders, M. J. N. L., Anbeek, P., Van Haastert, I. C., De Vries, L. S., & Groenendaal, F. (2012). Cerebellar volume and proton magnetic resonance spectroscopy at term, and neurodevelopment at 2 years of age in preterm infants. *Developmental Medicine and Child Neurology*, *54*(3), 260–266. https://doi.org/10.1111/j.1469-8749.2011.04168.x

Xu, D., Bonifacio, S., Charlton, N. N., Vaughan, C. P., Lu, Y., Ferriero, D. M., Vigneron, D. B., & Barkovich, A. J. (2011). MR Spectroscopy of Normative Premature Newborns. *Journal of Magnetic Resonance Imaging*, *33*(2), 306–311. https://doi.org/10.1002/jmri.22460
