# Supplementary material for: Neonatal brain metabolite concentrations: Associations with age, sex, and developmental outcomes
Source: PLoS One. 2020 Dec 17;15(12):e0243255. doi: 10.1371/journal.pone.0243255 (PMC7746171; doi:10.1371/journal.pone.0243255)
Supplement: S2 Table — (DOCX) [file pone.0243255.s002.docx]

**S2 Table. Associations of neonatal brain metabolite concentrations with later developmental outcomes**

| **Authors** | **Postnatal age (months) of developmental assessment** | **Developmental measures** | **Results** |
| --- | --- | --- | --- |
| Augustine et al. (2008) | 18 and 24, adjusted age | Bayley-II | No significant associations |
| Bapat et al. (2014) | 18 and 22, adjusted age | Bayley-III | Higher NAA/choline in subventricular zone and frontal cortex significantly associated with higher Bayley scores |
| Chau et al. (2013) | 18, adjusted age | Bayley-III | Slower increases in NAA/choline in gray and white matter were associated with worse outcomes |
| Gadin et al. (2012) | 6, adjusted age | Bayley-III | NAA/choline did not predict motor performance |
| Hart et al. (2014) | 18, adjusted age | Bayley-III, Amiel-Tison neuromotor examination | Higher NAA/choline and NAA/creatine in posterior white matter in those without neurodevelopmental difficulties compared to those with moderate or severe difficulties |
| Hyodo et al. (2018) | 18, adjusted age | Kyoto Scale | Mild developmental delay group showed a lower NAA/Cho ratio in the right and left thalamus and a lower NAA/Cr ratio in the left frontal white matter than the normal development group |
| Kendall et al. (2014) | 12, adjusted age | Bayley-III | Decreased NAA/choline and increased choline/creatine ratio predicted worse motor outcomes |
| Lally et al. (2019) | 18-24 | Bayley-III | Higher NAA associated with higher cognitive, language, and motor scores |
| Taylor et al. (2018) | 48 | WPPSI-III, CELF-Pre-2, Beery-Buktenica Test of Visual Motor Integration | No effects for neonatal metabolites or their rate of change |
| Van Kooij et al. (2012) | 24, adjusted age | Bayley-III | Higher NAA/choline ratio in cerebellum significantly associated with higher cognitive scores |

*Note*. See Table S1 for details of the methods used in these studies. Bayley-II, Bayley Scales of Infant Development, Second Edition; Bayley-III, Bayley Scales of Infant and Toddler Development, Third Edition; WPPSI-III, Wechsler Preschool and Primary Scale of Intelligence, Third Edition; CELF-Pre-2, Clinical Evaluation of Language Fundamentals-Preschool-2

**References**

Augustine, E. M., Spielman, D. M., Barnes, P. D., Sutcliffe, T. L., Dermon, J. D., Mirmiran, M., Clayton, D. B., & Ariagno, R. L. (2008). Can magnetic resonance spectroscopy predict neurodevelopmental outcome in very low birth weight preterm infants? *Journal of Perinatology*, *28*(9), 611–618. https://doi.org/10.1038/jp.2008.66

Bapat, R., Narayana, P. A., Zhou, Y., & Parikh, N. A. (2014). Magnetic Resonance Spectroscopy at Term-Equivalent Age in Extremely Preterm Infants: Association With Cognitive and Language Development. *Pediatric Neurology*, *51*(1), 53–59. https://doi.org/10.1016/j.pediatrneurol.2014.03.011

Chau, V., Synnes, A., Grunau, R. E., Poskitt, K. J., Brant, R., & Miller, S. P. (2013). Abnormal brain maturation in preterm neonates associated with adverse developmental outcomes. *Neurology*, *81*(24), 2082–2089. https://doi.org/10.1212/01.wnl.0000437298.43688.b9

Gadin, E., Lobo, M., Paul, D. A., Sem, K., Steiner, K. V., Mackley, A., Anzilotti, K., & Galloway, C. (2012). Volumetric MRI and MRS and early motor development of infants born preterm. *Pediatric Physical Therapy*, *24*(1), 38–44. https://doi.org/10.1097/PEP.0b013e31823e069d

Hart, A. R., Smith, M. F., Whitby, E. H., Alladi, S., Wilkinson, S., Paley, M. N., & Griffiths, P. D. (2014). Diffusion-weighted imaging and magnetic resonance proton spectroscopy following preterm birth. *Clinical Radiology*, *69*(8), 870–879. https://doi.org/10.1016/j.crad.2014.04.001

Hyodo, R., Sato, Y., Ito, M., Sugiyama, Y., Ogawa, C., Kawai, H., Nakane, T., Saito, A., Hirakawa, A., Kidokoro, H., Natsume, J., & Hayakawa, M. (2018). Magnetic resonance spectroscopy in preterm infants: Association with neurodevelopmental outcomes. *Archives of Disease in Childhood. Fetal and Neonatal Edition*, *103*(3), F238–F244. https://doi.org/10.1136/archdischild-2016-311403

Kendall, G. S., Melbourne, A., Johnson, S., Price, D., Bainbridge, A., Gunny, R., Huertas-Ceballos, A., Cady, E. B., Ourselin, S., Marlow, N., & Robertson, N. J. (2014). White matter NAA/Cho and Cho/Cr ratios at MR spectroscopy are predictive of motor outcome in preterm infants. *Radiology*, *271*(1), 230–238. https://doi.org/10.1148/radiol.13122679

Lally, P. J., Montaldo, P., Oliveira, V., Soe, A., Swamy, R., Bassett, P., Mendoza, J., Atreja, G., Kariholu, U., Pattnayak, S., Sashikumar, P., Harizaj, H., Mitchell, M., Ganesh, V., Harigopal, S., Dixon, J., English, P., Clarke, P., Muthukumar, P., … Thayyil, S. (2019). Magnetic resonance spectroscopy assessment of brain injury after moderate hypothermia in neonatal encephalopathy: A prospective multicentre cohort study. *The Lancet. Neurology*, *18*(1), 35–45. https://doi.org/10.1016/S1474-4422(18)30325-9

Taylor, M. J., Vandewouw, M. M., Young, J. M., Card, D., Sled, J. G., Shroff, M. M., & Raybaud, C. (2018). Magnetic resonance spectroscopy in very preterm-born children at 4 years of age: Developmental course from birth and outcomes. *Neuroradiology*, *60*(10), 1063–1073. https://doi.org/10.1007/s00234-018-2064-7

Van Kooij, B. J. M., Benders, M. J. N. L., Anbeek, P., Van Haastert, I. C., De Vries, L. S., & Groenendaal, F. (2012). Cerebellar volume and proton magnetic resonance spectroscopy at term, and neurodevelopment at 2 years of age in preterm infants. *Developmental Medicine and Child Neurology*, *54*(3), 260–266. https://doi.org/10.1111/j.1469-8749.2011.04168.x
