# Supplementary figures and images for: Neonatal brain metabolite concentrations: Associations with age, sex, and developmental outcomes
Source: PLoS One. 2020 Dec 17;15(12):e0243255. doi: 10.1371/journal.pone.0243255 (PMC7746171; doi:10.1371/journal.pone.0243255)

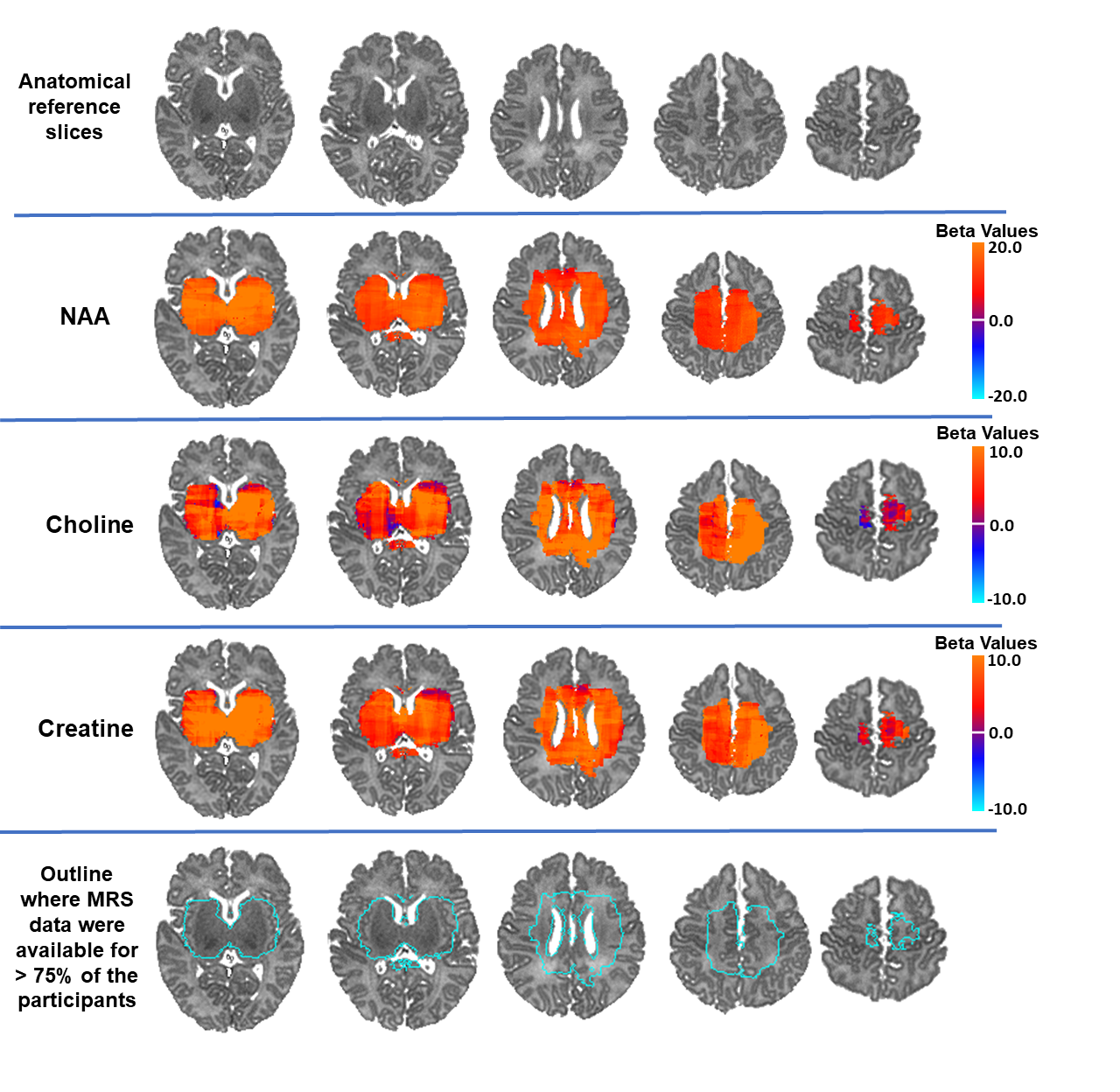

Supplement: S1 Fig — The magnitude of association is displayed in terms of beta values from multiple linear regression applied voxel-wise throughout the brain separately for each of the three metabolite values (NAA, Ch, and Cr) entered as the dependent variable and PMA at scan and sex entered simultaneously as independent variables. Anatomical reference slices are presented in the top row. Maps indicating the voxels for which at least 75% of the sample had usable data are presented in the bottom row. (TIF) [file pone.0243255.s003.tif]

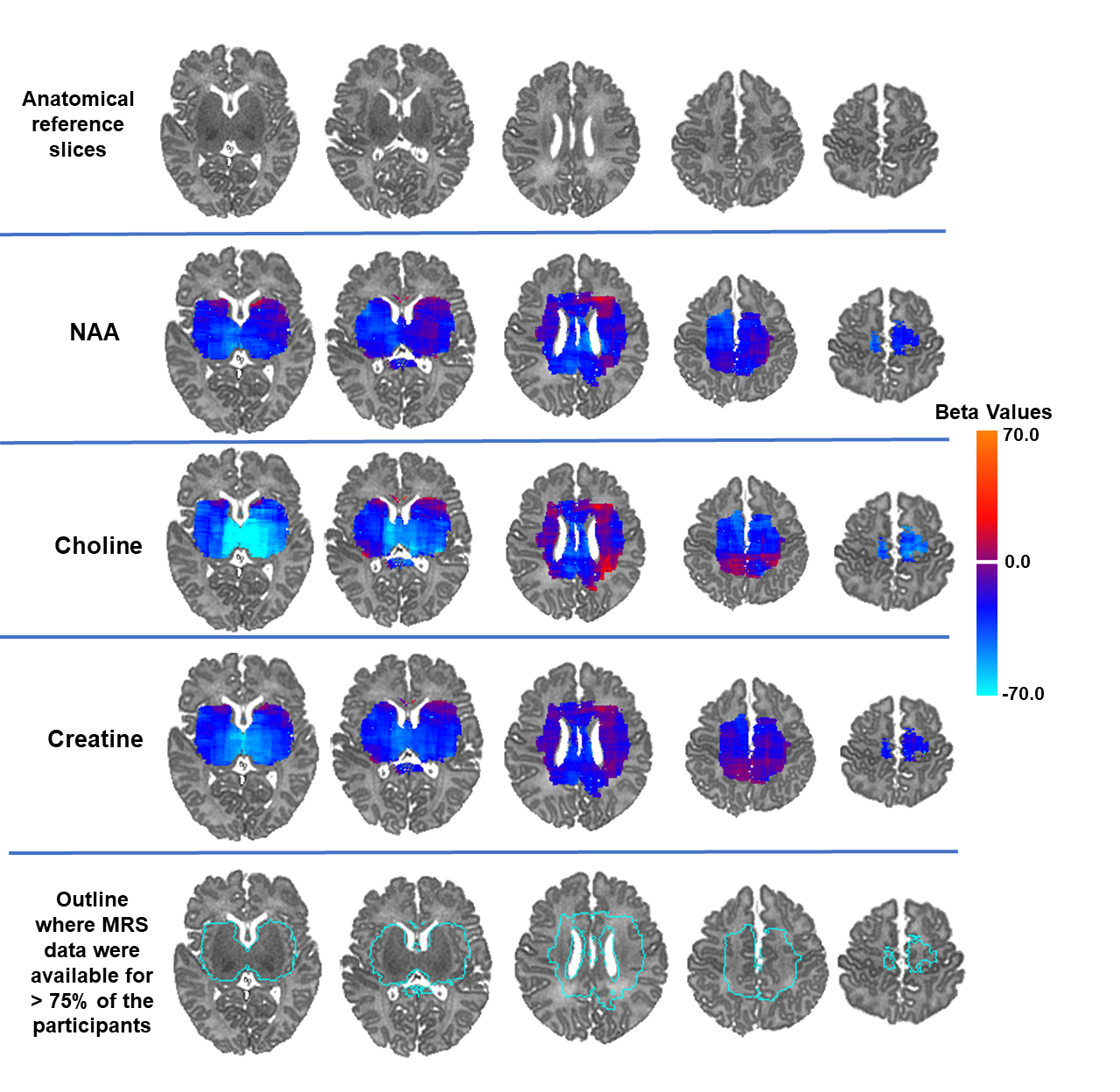

Supplement: S2 Fig — The magnitude of the association is displayed in terms of beta values from multiple linear regression applied voxel-wise throughout the brain separately for each of the three metabolite values (NAA, Ch, and Cr) entered as the dependent variable and PMA at scan and sex entered simultaneously as independent variables. Anatomical reference slices are presented in the top row. Maps indicating the voxels for which at least 75% of the sample had usable data are presented in the bottom row. (TIF) [file pone.0243255.s004.tif]

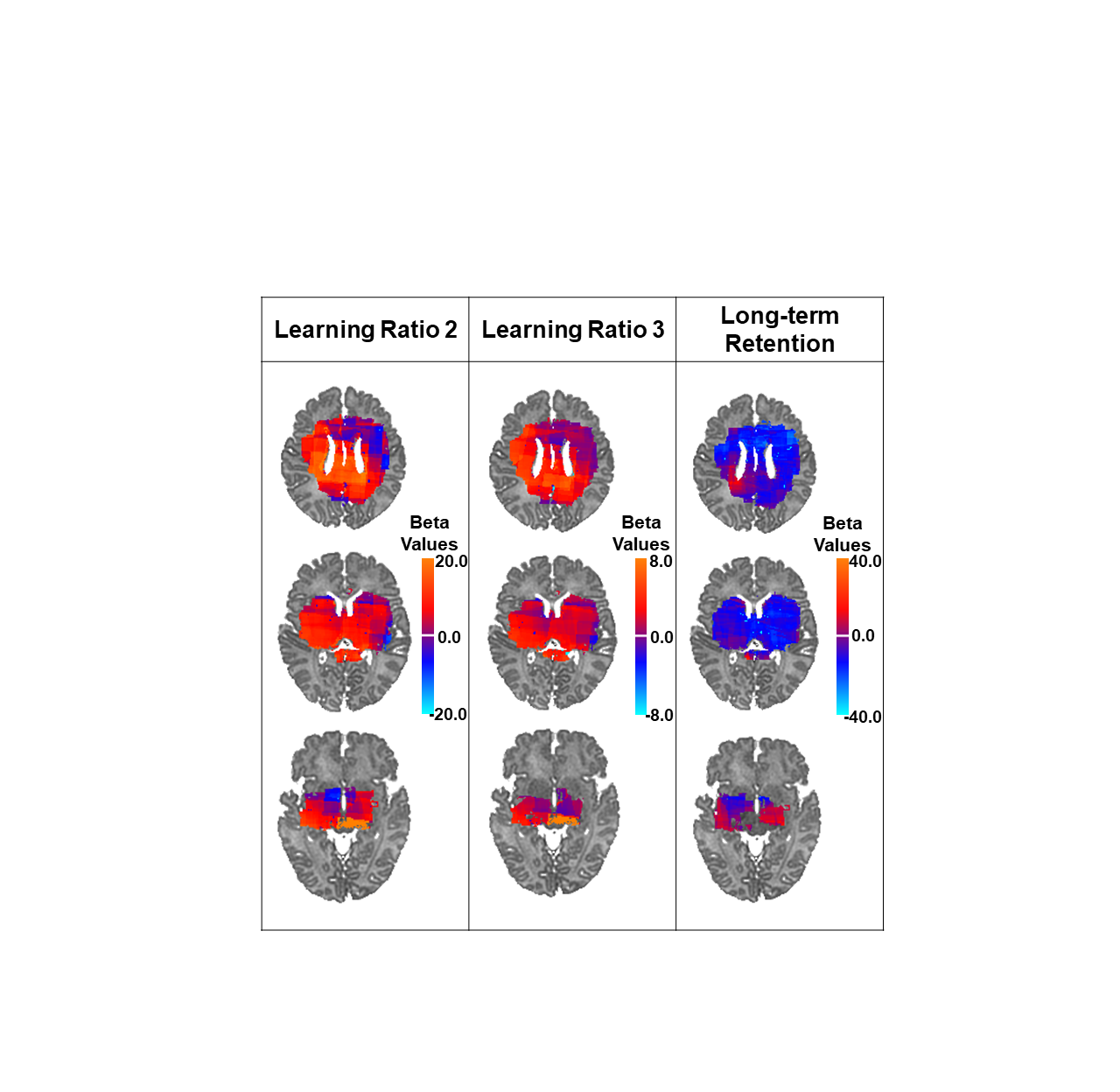

Supplement: S3 Fig — Beta values are displayed from multiple linear regression applied voxel-wise throughout the brain for NAA entered as the dependent variable and ratio score as the independent variable, with PMA and sex included as covariates. Brain maps are presented for ratio scores on learning block 2, learning block 3, and the long-term retention block (Day 2 baseline) for the mobile conjugate reinforcement paradigm. (TIF) [file pone.0243255.s005.tif]
