# Supplementary material for: Neonatal brain metabolite concentrations: Associations with age, sex, and developmental outcomes
Source: PLoS One. 2020 Dec 17;15(12):e0243255. doi: 10.1371/journal.pone.0243255 (PMC7746171; doi:10.1371/journal.pone.0243255)
Supplement: S1 Text — (DOCX) [file pone.0243255.s006.docx]

**Sample Ascertainment and Attrition**

Data used in this study came from a larger study of healthy, nulliparous pregnant adolescents and young adults who were actively engaged in prenatal care. Of 324 pregnant adolescents enrolled in this larger study, 154 who enrolled later in the study were offered newborn MRI scans and infancy follow-up sessions. Of these 154, 82 newborns underwent MRI scans. Reasons for not acquiring newborn MRI data were the following: withdrew from the study (*n* = 18), declined MRI scan during the pilot portion of the study (*n* = 6), consented to the MRI scan but decided not to during the MRI session (*n* = 12), unable to be scheduled (*n* = 18), technical problems with scanner (*n* = 9), not available on day of scheduled scan (*n* = 8), baby in NICU (*n* = 1). MPCSI data were acquired in 41 newborns. This sequence was subsequently discontinued at our location, precluding scanning more infants. The MRI scanner was upgraded and the new scanner was not compatible with the MPCSI pulse sequence.

Of the 41 infants with MPCSI data, 27 had developmental outcome (Bayley-III and/or mobile conjugate reinforcement paradigm) data at 4 months. Those with developmental data at 4 months did not differ significantly from those without these data in terms of infant gestational age at birth (*p* = .57), infant sex (*p* = .46), maternal race (*p* = .16), or maternal ethnicity (*p* = .22). However, infants with 4-month developmental data had mothers who were younger than those of infants without these data, *t*(39) = 2.31, *p* = .03.

**Processing of Multiplanar Chemical Shift Imaging (MPCSI) Data**

MRS data quality was assured by reconstructing the data, assessing it for excess noise, and examining the spectrum in each voxel for baseline distortions, signal contamination by lipid signal from the scalp, incorrect placement of suppression bands, or broadening of line width. MRS voxels containing any of these artifacts were not processed further. More specifically, first, we loaded the MPCSI data into the software platform 3DiCSI (3D Interactive Chemical Shift Imaging) and visually assessed and eliminated any MRS voxel that showed strong lipid signal contamination or that contained insufficiently suppressed residual water signal. Second, our automated spectral fitting algorithm removed any voxel in which the fitted creatine (Cr), choline (Ch), or N-acetylaspartate (NAA) peak had a full width at half maximum > 12 Hz. Subsequently, we visually assessed the fitted spectrum to detect the presence of unresolved Cr and Ch peaks. Any MRS voxel that contained unresolved Cr and Ch peaks were deleted from further processing. Thus, we analyzed MRS data from only those voxels in the brain that were not contaminated with lipid signal, that had narrow metabolite peaks that were clearly resolved from other peaks, and in which water signal was suppressed.

MPCSI provides metabolite data across the entire brain and, therefore, allows comparison of metabolite data at homologous brain regions across participants. Furthermore, MPSCI data permitted us to correct for partial volume effects of different tissue types that contribute to MRS signal in each voxel.

We processed the signal from each coil of the 8-channel head coil separately before combining their processed MRS signals to generate the spectroscopic images (Dong & Peterson, 2007). First, we phase-aligned signals, then smoothed the aligned signals using a Hamming window filter, spatially reconstructed the time-domain free induction decay (FID) signal in each slice with a 2D Fourier transform, suppressed residual water signal by applying a high pass filter to the FID signal, performed line broadening using a 4 Hz Gaussian filter, and then transferred the time-domain signal into the frequency domain with a 1D Fourier transform (Hao et al., 2013). Finally, the processed frequency-domain signal from each of the 8 coils was combined by computing their weighted sum. The combined signal was then loaded into the software *3DiCSI* (3D Interactive Chemical Shift Imaging) to identify MRS voxels within the brain and save spectral data for those voxels.

We used model-based spectral fitting to model the spectrum in each voxel with Voigtian curves to the peaks for NAA, Cr, and Cho. The area under the peaks for each of the three metabolites was obtained by integrating the corresponding fitted lines. As described above, MRS voxels with (a) lipid contamination, (b) insufficient suppression of residual water, (c) unresolved Cr and Ch peaks, or (d) a full width at half maximum >12 Hz for any peaks were rejected and not processed any further. To account for variations in receiver gain (RG), we conducted a series of phantom scans with increasing RGs and calculated the ratio of peak area to the noise level for each RG. We used these ratios as correction factors to compensate for the effects of varying RGs on peak areas. Effects of transmitter gains (TG) were also corrected against an arbitrary value of TG_0_ =15.6 dB according to$S=S_{0}*{10}^{(TG-TG_{O})/20}$. Background noise was calculated as the standard deviation of the real part of the complex data in the regions free of signal from metabolites. We then computed the signal-to-noise ratio (SNR) for each metabolite level, which were subsequently used in all analyses. The average signal-to-noise ratio (SNR) of NAA, defined as the peak area of NAA to the standard deviation of data in the signal-free region of the spectrum, was greater than 35, an excellent SNR attributable to use of the multichannel coil.

A spectroscopic image for each metabolite was generated next as the ratio of the peak area to the background noise for each MRS voxel within the brain. We used the ratio of the peak area to the background noise as our dependent measure when assessing the age correlates of each metabolite concentration because it accounted for variations in receiver and transmitter gain within the MRS data across participants. Because we suppressed water signal, we were unable to calculate metabolite ratios using water as the reference. We elected to measure each neurometabolite individually, rather than as a ratio to creatine (a common practice in spectroscopy research), for several reasons. First, ratios accentuate noise in the numerator and denominator and are generally undesirable for statistical analyses. Second, ratios may lead to erroneous inferences in developmental studies because both the numerator and denominator can vary with age, in which case interpreting the age correlates of the ratio requires that the effects of the numerator and denominator be assessed separately to understand which metabolite is driving the ratio findings. Our use of metabolite concentrations normalized to noise values overcomes these difficulties and provides a much clearer understanding of the age correlates of brain metabolite levels.

Partial Volume Correction In addition to these standard MPCSI data processing steps, we performed partial volume correction on the NAA values. Partial volume effects are pronounced in MPCSI data and would limit the accuracy of correlations with data in other modalities. Two sources contribute to MPCSI partial volume effects. A large spectroscopic imaging voxel usually consists of varying proportions of brain tissue and CSF. In addition, the limited number of k-space sampling points in MPCSI bleeds signal across voxels, termed a “point-spread-function” (PSF) effect, which must be taken into account when correcting for partial volume effects. The PSF of an MRSI signal is a complex function that describes how the MR signal from one voxel spreads to other voxels over the entire field of view. The PSF is determined in part by the k-space trajectory during data acquisition. It is also determined by the window function for spatial filtering employed prior to Fourier reconstruction to suppress long range signal bleeding, but at the price of increasing signal contamination across adjacent voxels. We calculated the PSF by simulating the MRSI acquisition in an inscribed circle of 16x16 grids in k-space and subsequently spatial filtering the data with a Hamming window function. The resulting 16x16 complex array was interpolated to 256x256 to match the high resolution MR images. To obtain the compartment images with the same resolution and PSF effect as the MPCSI, we segmented the high-resolution T2-weighted MR images that were coregistered to the MPCSI slices into components of brain tissue and CSF. We then convolved them with the PSF.

From these low-resolution compartment images, we retrieved the NAA concentrations from the brain tissue and CSF in the *i*-th voxel using a linear regression model (Lebon et al., 2002): $S_{i}=\sum_{b} |c_{b}^{i}M_{b}|+n$, where S_i_ is the measured data for the metabolite, $c_{b}^{i}$is the point spread structural representation of brain tissue, $M_{b}$ is the tissue contribution to the metabolite signal, and *n* is noise. The partial volume-corrected tissue metabolite concentrations at the resolution of the MRS data are then assigned to corresponding tissue voxels at a spatial resolution of 1x1x1 mm^3^ of the anatomical MR image.

Spatial Normalization of MPCSI Data MPCSI data for each participant were coregistered into the coordinate space of a T2-weighted image of the template brain. Specifically, each participant’s localizer image was coregistered to its high-resolution T2w image using a similarity transformation (3 translations and 3 rotations) such that the transformation maximized mutual information (Viola & Wells, 1995) across the localizer and its corresponding high resolution T2w image. Second, we spatially transformed the localizer image using the similarity transformation that coregistered the T2w image of the participant into the coordinate space of the template brain. Third, we warped the coregistered localizer by applying to it the high-dimension, nonlinear deformation that warped the participant T2w image to the template T2w image. We applied these 3 coregistration procedures to each of the metabolite images.

The MPCSI saturation bands applied to suppress lipid signal from the scalp were not as precisely shaped as the scalp, and they unavoidably suppressed metabolite signals from several portions of cortical gray matter. Moreover, lipid signal from the small portions of scalp that were unsuppressed contaminated MRS signal to some degree within the brain, and those voxels were censored from further analyses during the detailed visual inspection of the spectra. Consequently, metabolite measures for many participants were available only in voxels of white matter and deep gray matter nuclei. *We therefore presented results at only those voxels that had usable data from 75% or more of the infants.*

**Selection of the Template Brain**

We used a single representative brain for the template, rather than one derived by averaging brains across multiple infants, because a single brain has well-defined tissue interfaces, including those at CSF-gray matter or gray-white matter interfaces, that improve the precision of spatial co-registration and the identification of corresponding points across brains. Moreover, gyri and sulci on the cerebral surface differ markedly across individuals,^15^ and not all individuals have a particular gyrus or sulcus, complicating the generation of a synthetic, average cerebral surface.

We employed a rigorous, 2-step procedure to select a template brain to ensure that findings were not unduly influenced by selection of a non-representative template. First, using the T2-weighted anatomical images for each infant, we identified as a preliminary template the brain of one infant whose PMA and overall brain size were nearest the group averages. The brains for all remaining unexposed infants in the sample were coregistered to that preliminary template, and then the distance of each point on the surface of each brain was measured from the corresponding point on the preliminary template surface. The brain for which all points across its surface were closest (in the least squares sense) to the average of the distances across those points for the entire sample was selected as the final template, thereby yielding a template brain that is specific to and morphologically most representative of all infant brains in this cohort. Despite the care taken in selection of the most representative template brain, we also note that our findings are robust with respect to the specific template used, as the findings generated when using randomly selected brains from our sample as the template differed minimally from those generated using the most representative brain as the template.

**Bayley Scales of Infant and Toddler Development-Third Edition (Bayley-III)**

The Bayley-III (Bayley, 2005) Cognitive, Language and Motor scales were used in this study. The Cognitive scale assesses sensorimotor development, exploration and manipulation, object relatedness, concept formation, memory, and simple problem solving. The Language scale consists of the Receptive Communication (verbal comprehension, vocabulary) and Expressive Communication (babbling, gesturing, and utterances) subtests. The Motor scale consists of the Fine Motor (grasping, perceptual-motor integration, motor planning, and speed) and Gross Motor (sitting, standing, locomotion, and balance) subtests. Raw and age-standardized scores (*M* = 100, *SD* = 15) for the Cognitive, Language, and Motor scales were calculated. The Bayley-III has acceptable psychometric properties (Cronbach’s *α* = .91-.93; six-day test-retest reliability = .80) and norms based on a racially- and socioeconomically-representative sample (Albers & Grieve, 2007), although some limitations of this measure have been noted (Anderson et al., 2010; Anderson & Burnett, 2017; Moore et al., 2012).

**Mobile Conjugate Reinforcement Paradigm**

At 4-months postpartum, mothers brought their infants to the lab on two consecutive days for participation in the mobile conjugate reinforcement paradigm. Following standard procedures (Merz et al., 2017; Rovee-Collier et al., 1999), the infant was first placed inside a seat located inside a plain wooden crib. Hanging over the crib from one of two L-shaped metal brackets was a mobile with four brightly painted animal toys hanging from strings. One end of a ribbon was tied around the infant's left or right ankle and the other end was attached to one of the metal brackets. Each videotaped 15-min session began with a 3-min non-reinforcement phase (baseline), followed by a 9-min reinforcement phase (three 3-min learning blocks), and a final 3-min non-reinforcement phase (immediate retention). During periods of non-reinforcement, the ribbon was attached to the metal bracket without the mobile hanging from it. In this arrangement, any movement of the leg with the ribbon on it had no effect on the mobile. During periods of reinforcement, the ribbon was attached to the metal bracket with the mobile hanging from it. Thus, kicking the leg with the ribbon attached to it caused the mobile to bounce. On Day 1, the initial 3-min period of non-reinforcement (baseline) provides a measure of the infant's baseline kick rate, and the final 3-min period of non-reinforcement provides a measure of the infant's immediate retention. On Day 2, kicking during the initial period of non-reinforcement (Day 2 baseline) reflects the infant's long-term (24-hr) retention of the contingency.

Trained coders used the videotapes to count the number of times per minute infants kicked the leg with the ribbon attached to it. A kick was defined as a linear or circular movement of the foot and leg retraced in a continuous motion back to the point of origin (Rovee-Collier et al., 1999). A second trained coder independently coded 20% of the sessions. Inter-rater reliability was high (Spearman rank correlation = .95; range: .88–.99). The baseline ratio was calculated by dividing the mean kick rate during each learning and retention block (immediate retention, long-term retention) by the mean kick rate during Day 1 baseline.

We observed some large values for the learning block ratio scores, and the ratio score data were skewed, consistent with previous research (Haley et al., 2006; Merz et al., 2017; Rovee-Collier et al., 1999; Rubin et al., 1998; Schroers et al., 2007). More specifically, two values for learning block 2 and learning block 3 raised concern for being outliers. Although excluding the large values for learning block 2 did not change the results for learning block 2, excluding the two large learning block 3 ratio scores from the analyses did change the results for learning block 3. These learning block ratio score data are unlikely due to measurement error and therefore provide important information about the associations between brain metabolite concentrations and learning outcomes. Large learning block ratio scores likely indicate an infant kicking frequently during a learning block but infrequently during baseline, as described in previous research (Haley et al., 2006).

**References**

Albers, C. A., & Grieve, A. J. (2007). Review of Bayley Scales of Infant and Toddler Development—Third Edition. *Journal of Psychoeducational Assessment*, *25*(2), 180–190. https://doi.org/10.1177/0734282906297199

Anderson, P. J., & Burnett, A. (2017). Assessing developmental delay in early childhood—Concerns with the Bayley-III scales. *The Clinical Neuropsychologist*, *31*(2), 371–381. https://doi.org/10.1080/13854046.2016.1216518

Anderson, P. J., De Luca, C. R., Hutchinson, E., Roberts, G., Doyle, L. W., & Victorian Infant Collaborative Group. (2010). Underestimation of developmental delay by the new Bayley-III Scale. *Archives of Pediatrics & Adolescent Medicine*, *164*(4), 352–356. https://doi.org/10.1001/archpediatrics.2010.20

Dong, Z., & Peterson, B. (2007). The rapid and automatic combination of proton MRSI data using multi-channel coils without water suppression. *Magnetic Resonance Imaging*, *25*(8), 1148–1154. https://doi.org/10.1016/j.mri.2007.01.005

Haley, D. W., Weinberg, J., & Grunau, R. E. (2006). Cortisol, contingency learning, and memory in preterm and full-term infants. *Psychoneuroendocrinology*, *31*(1), 108–117. https://doi.org/10.1016/j.psyneuen.2005.06.007

Hao, X., Xu, D., Bansal, R., Dong, Z., Liu, J., Wang, Z., Kangarlu, A., Liu, F., Duan, Y., Shova, S., Gerber, A. J., & Peterson, B. S. (2013). Multimodal Magnetic Resonance Imaging: The Coordinated Use of Multiple, Mutually Informative Probes to Understand Brain Structure and Function. *Human Brain Mapping*, *34*(2), 253–271. https://doi.org/10.1002/hbm.21440

Lebon, V., Petersen, K. F., Cline, G. W., Shen, J., Mason, G. F., Dufour, S., Behar, K. L., Shulman, G. I., & Rothman, D. L. (2002). Astroglial Contribution to Brain Energy Metabolism in Humans Revealed by 13C Nuclear Magnetic Resonance Spectroscopy: Elucidation of the Dominant Pathway for Neurotransmitter Glutamate Repletion and Measurement of Astrocytic Oxidative Metabolism. *The Journal of Neuroscience*, *22*(5), 1523–1531.

Merz, E. C., McDonough, L., Huang, Y. L., Foss, S., Werner, E., & Monk, C. (2017). The mobile conjugate reinforcement paradigm in a lab setting. *Developmental Psychobiology*, *59*(5), 668–672. https://doi.org/10.1002/dev.21520

Moore, T., Johnson, S., Haider, S., Hennessy, E., & Marlow, N. (2012). Relationship between test scores using the second and third editions of the Bayley Scales in extremely preterm children. *The Journal of Pediatrics*, *160*(4), 553–558. https://doi.org/10.1016/j.jpeds.2011.09.047

Rovee-Collier, C., Hartshorn, K., & DiRubbo, M. (1999). Long-term maintenance of infant memory. *Developmental Psychobiology*, *35*(2), 91–102.

Rubin, G. B., Fagen, J. W., & Caroll, M. H. (1998). Olfactory context and memory retrieval in 3-month-old infants. *Infant Behavior and Development*, *21*(4), 641–658. https://doi.org/10.1016/S0163-6383(98)90035-2

Schroers, M., Prigot, J., & Fagen, J. (2007). The Effect of a Salient Odor Context on Memory Retrieval in Young Infants. *Infant Behavior & Development*, *30*(4), 685–689. https://doi.org/10.1016/j.infbeh.2007.05.001

Viola, P., & Wells, W. (1995, June 20). *Alignment by Maximization of Mutual Information*. IEEE Proc. of the 5th Int. Conf. on Computer Vision, Boston, MA.
